# Functional Mapping of Key Residues in Reductive Aminases Enabled by a High-Throughput *RedAm Detect* Assay

**DOI:** 10.1021/jacsau.5c00512

**Published:** 2025-06-27

**Authors:** Jinming Xing, Georgie Orderley, Ruth T. Bradshaw Allen, Nabieha N. Ahmad, Camille Gourjault, Ardil Akgul, Sephia O. Alhassan, Nichapa Ngernanek, Siddhika Salke, Godwin A. Aleku

**Affiliations:** Institute of Pharmaceutical Science, Franklin-Wilkins Building, 4616 King’s College London, 150 Stamford Street, London SE1 9NH, United Kingdom

**Keywords:** high-throughput screening, reductive amination, imine reductases, reductive aminases, amino acid dehydrogenases, amine dehydrogenases, amine oxidases, sequence-activity relationship

## Abstract

Enzymatic reductive amination allows direct and stereoselective access to 1°, 2°, and 3° chiral amines under environmentally friendly reaction conditions. Enzyme discovery and engineering campaigns for this important transformation are crucial for industrial applications but currently rely on tedious and time-consuming screening of large libraries using expensive LC/LC-MS systems. Such engineering campaigns have also focused on optimizing a single aminase candidate per defined synthetic target. In this work, we have developed a versatile high-throughput (HTP) spectrophotometric/colorimetric *RedAm detect* assay for rapid and reliable quantitative monitoring of the aminase activity of recombinant aminase-expressing cells or unpurified cell extracts. The assay couples aminase product formation to an amine oxidase–HRP reporter system, yielding a colored dye/signal that can be monitored at 492/498 nm. We demonstrate its application to quantitatively monitor reductive amination reactions catalyzed by reductive aminases (RedAms), amine dehydrogenases (AmDHs), and amino acid dehydrogenases (AADHs). The *RedAm detect* assay enabled the HTP screening of 56 site saturation libraries in two RedAms, revealing 10 positions, including N113, D135, D188, Y196, W227, T234, Q257, A262, S270, and D297 in *Bac*RedAm as important residues for RedAms’ catalytic function/substrate specificity. Extending our screening to four substrate combinations enabled the identification of positions R53, T115, Y154, L189, M195, Y196, W227, and Q257 in *Bac*RedAm (and equivalent positions: R35, T99, Y139, L174, M180, Y181, W211, and Q241 in *Ma*RedAm) that yielded mutants with improved activity of up to 7-fold compared to the wild-type enzyme in at least three of the four transformations. We propose that these residues act as potential “universal” hotspots for engineering substrate specificity in these enzymes across diverse substrates. Our work lays an important foundation for mapping sequence-activity relationships in RedAms at the enzyme family level to pave the way for more predictable, faster, and cost-effective engineering of RedAms for applications.

## Graphical abstract

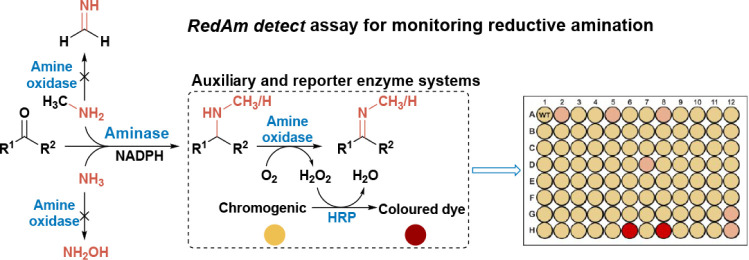

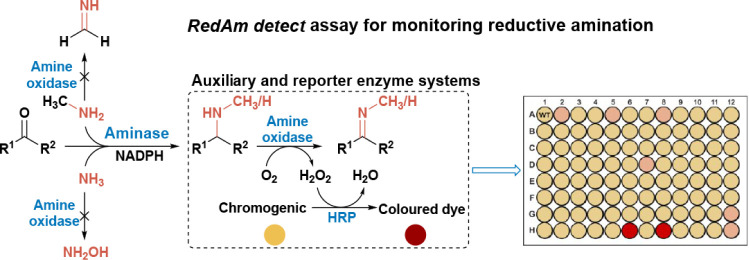

## Introduction

Reductive amination (RA) of carbonyl compounds is among the most widely employed transformations in the synthesis of active pharmaceutical ingredients (APIs).^1^ Despite its widespread use, achieving green and enantioselective versions of this transformation remains a significant challenge.^2^ In recent years, enzyme-catalyzed reductive amination has emerged as one of the most promising enantioselective amination approaches, offering high enantioselectivity under mild and environmentally friendly conditions.

An expanding repertoire of NAD­(P)­H-dependent aminases, such as reductive aminases (RedAms),^3–8^ amine dehydrogenases (AmDHs),^9,10^ and (*N*-alkyl)­amino acid dehydrogenases ((NA)­AADH_S_),^11,12^ enables direct and selective access to primary (1°) chiral amines as well as secondary (2°) and tertiary (3°) chiral amines without the need for further *N*-alkylation step(s) and under mild reaction conditions. However, enzyme engineering campaigns aimed at improving the efficiency of these enzymes for industrial application have typically focused on optimizing preselected aminase candidates toward defined synthetic targets,^4–7^ with little or no exploration of family level sequence-activity relationships.

Functional mapping of sequence-function relationships established for multiple members can reveal “universal” hotspots in an aminase enzyme family to facilitate speedy and cost-effective engineering of a member of that family for application. Inspired by our ambition to contribute toward expanding the repertoire of aminating enzymes^13^ and the elucidation of family level sequence-activity relationship patterns in RedAms, we aimed to (i) develop a versatile high-throughput colorimetric assay that enables the detection of aminase activity in cell lysates and (ii) map the functional roles of multiple conserved residues in two RedAms to pinpoint key residues contributing to substrate specificity and RedAms’ catalysis.

We first sought to develop a novel spectrophotometric/colorimetric high-throughput *RedAm detect* assay for rapid and reliable quantitative monitoring of aminase activity in recombinant aminase-expressing cells or unpurified cell extracts. Such desirable rapid assays have often proven challenging to develop. The presence of endogenous carbonyl reductases from host cells, with their competing NAD­(P)­H-dependent carbonyl-reducing activity, diminishes the specificity of direct NAD­(P)H depletion assays and NADP­(H)-based coupled assays utilizing tetrazolium salts as intermediate hydrogen carriers. As such, these assays have primarily been applied to screen RedAms’ dehydrogenase activity in the oxidative direction, e.g., IREDy-to-go.^14^ Engineering campaigns focused on improving aminase activity have thus relied on time-consuming and expensive LC/LC-MS screening systems.^4–7,15^

To overcome these limitations, we developed a *RedAm detect* assay which monitors amine product formation rather than NAD­(P)­H depletion (Figure 1). The amine-forming step of RedAm/aminase-catalyzed reductive amination is coupled to an amine oxidase and H_2_O_2_-dependent reporter system, Figure 1a. This enables end point and kinetic measurement of RedAm activity, providing a robust, lysate-compatible alternative to time-intensive LC-based screening.

**Figure 1:**
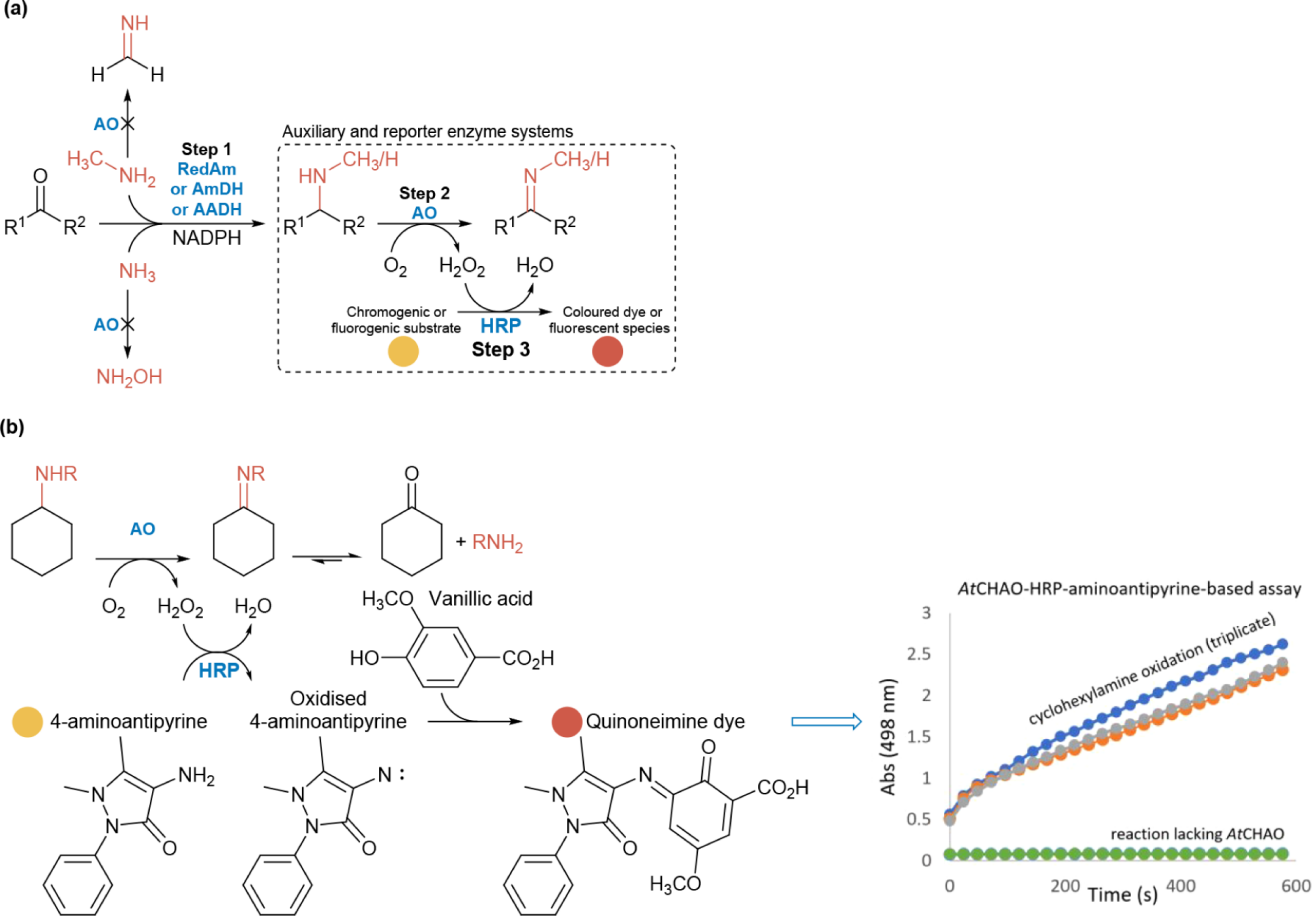
Design of the one-pot *RedAm detect* assay for monitoring enzymatic reductive amination reactions. (a) Schematic overview of the one-pot *RedAm detect* assay for monitoring reductive amination reactions. The amine product formed via an aminase-mediated catalysis (Step 1) is selectively oxidized in the downstream amine oxidase-H_2_O_2_–HRP systems which in turn mediates the condensation of the readily available 4-aminoantipyrine and vanillic acid to yield a red quinoneimine dye, enabling absorbance-based quantification at 498 nm (molar absorbance coefficient, *ε* = 6234 M^–1^ cm^–1^) using a microtiter plate reader. (b) An amine oxidase and H_2_O_2_-dependent reporter system coupled to the aminase step of *RedAm detect* assay. Ao, amine oxidase; *At*CHAO, cyclohexylamine oxidase from *Arthrobacter sp;* RedAm, reductive aminase; AmDH, amine dehydrogenase; AADH, amino acid dehydrogenase; HRP, horseradish peroxidase.

## Results and Discussion

### Amine Oxidases with Selective and Complementary Substrate Profiles for *RedAm Detect*

The *RedAm detect* assay relies on the amine oxidase selectively oxidizing the amine product (step 2, Figure 1a) formed from the amination reaction while remaining inert to the usually smaller amine nucleophiles (e.g., methylamine, ethylamine, and other “small” alkylamines) used as coupling partners in the RedAm step (step 1), Figure 1a. To ensure amine oxidases meet this criterion, we selected two versatile amine oxidases, cyclohexylamine oxidase from *Arthrobacter* *sp (At*CHAO),^16^ and MAON D9, a variant of monoamine oxidase from *Aspergillus niger*, previously shown to display a broad substrate scope.^17,18^ We investigated their substrate profile and reactivity pattern against a panel of 48 structurally diverse amine substrates **1**–**48** using a horseradish peroxidase (HRP)-H_2_O_2_ reporter system, as shown in Figure 2.

**Figure 2:**
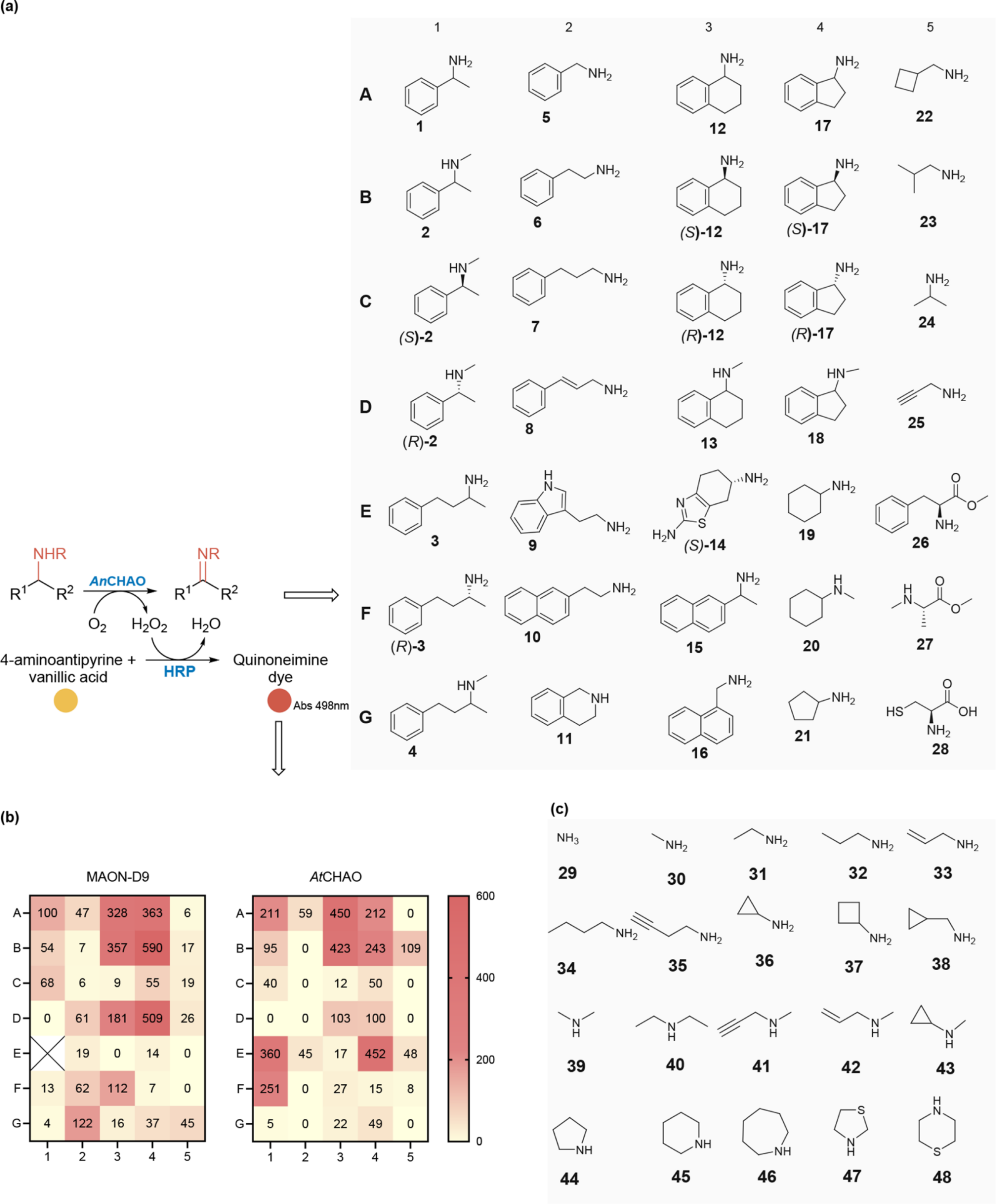
Substrate profiling of two versatile amine oxidases (MAON-D9 and *At*CHAO). (a) A panel of amine substrates screened against MAON-D9 and *At*CHAO and displayed detectable activity. (b) Heatmap displaying % relative specific activity of MAON-D9 and *At*CHAO toward the oxidation of a panel of amines. Specific activity of MAON-D9 toward amine **1**=1.2 U mg^–1^ was set as 100%. (c) A panel of amine substrates screened against MAON-D9 and *At*CHAO with no activity detected. Reaction conditions: 180 μL of the reaction buffer containing 1 U ml^–1^ HRP, 4 mM aminoantipyrine, 4 mM vanillic acid in sodium phosphate buffer (100 mM, pH 8) was added to each well of the microtiter plate. 5 μL (6.25 mM) of each amine substrate was added to a well from a stock solution in DMSO. The reaction was started with the addition of 15 μL purified amine oxidase, *At*CHAO or MAON-D9 (final concentration 0.3 mg mL^–1^) and monitored at 498 nm for 1 h using a microtiter plate reader. MAON D9, a variant of monoamine oxidase from *Aspergillus niger* *; AtCHAO,* cyclohexylamine oxidase from *Arthrobacter* *sp*.

Several primary amines **1**, **3**, **5**, **12**, **14**, **15**, **17**, **19**, **21**, and **23**, as well as their corresponding *N*-methyl analogues **2**, **4, 13**, **18**, and **20** showed good to moderate reactivity with *At*CHAO and/or MAON D9. When both (*S*) and (*R*) enantiopure amines were tested, e.g., (*R*)-**2** versus (*S*)-**2** or (*R*)-**12** versus (*S*)-**12**, the amine oxidases showed clear stereopreference for the (*S*)-configured amines, although in some cases, *At*CHAO displayed good or moderate activity toward some (*R*)-configured amines, e.g., (*R*)-**3** and (*R*)-**17**, Figure 2a,b. α-Amino esters/α-amino acids **26**–**28** were oxidized albeit with lower efficiency.

Crucially, small amine nucleophiles, such as ammonia **29**, methylamine **30**, and several smaller alkylamines **31**–**43**, as well as cyclic amines **44**–**48**, showed no detectable activity with *At*CHAO or MAON-D9 under assay conditions, Figure 2c. Mapping substrate specificity in this manner confirms that an *At*CHAO/MAON-D9-based reporter system can indeed be coupled to a RedAm-catalyzed amine-forming step in one pot, as *At*CHAO and MAON-D9 are highly selective for amine products of the RedAm-catalyzed reactions and remain inert toward the amine coupling partners, such as ammonia **29** and methylamine **30**, and several smaller alkylamines (Figure 2c) that are frequently employed as amine coupling partners in the RedAm step.^3^

### Novel *RedAm Detect* Assay for Rapid Monitoring of Reductive Aminase Activity of Cell Extracts

Having identified amine oxidases with selective and complementary substrate tolerance to RedAms, we aimed to integrate the RedAm and the downstream amine oxidase/HRP steps in a one-pot assay format, as shown in Figure 3. This would allow for a direct and reliable photometric readout of RedAm activity. Initially, we examined the reductive amination of cyclohexanone **49** with methylamine **30** and NADPH as a model transformation. Two reaction buffers were formulated: buffer A1, containing 10 mM cyclohexanone **49**, 40 mM methylamine **30**, and 2 mM NADPH in 100 mM Tris pH 8.5; and buffer B1, which contained all components to drive the amine oxidase and the downstream HRP reactions toward the formation of the red quinoneimine dye, excluding the amine substrate. To assess the *RedAm detect* as an end point assay, the RedAm-catalyzed reductive amination was first performed using buffer A1 for a set incubation time, e.g., 1 h. When assessing full conversion, NADPH was supplied at a 1.2 molar ratio to the carbonyl, or an NADP­(H) recycling system was used. After incubation, buffer B1 was added to initiate the downstream reaction (prepared in 100 mM Tris pH 7.5). A bacterial reductive aminase, *Bac*RedAm,^19^ was used as the prototype RedAm catalyst. The formation of a red dye was promptly observed upon adding buffer B1 to the *Bac*RedAm reaction and was absent in the control reaction which lacked *Bac*RedAm. The absorbance of the formed dye was monitored at 498 nm following an additional 10 min incubation.

**Figure 3:**
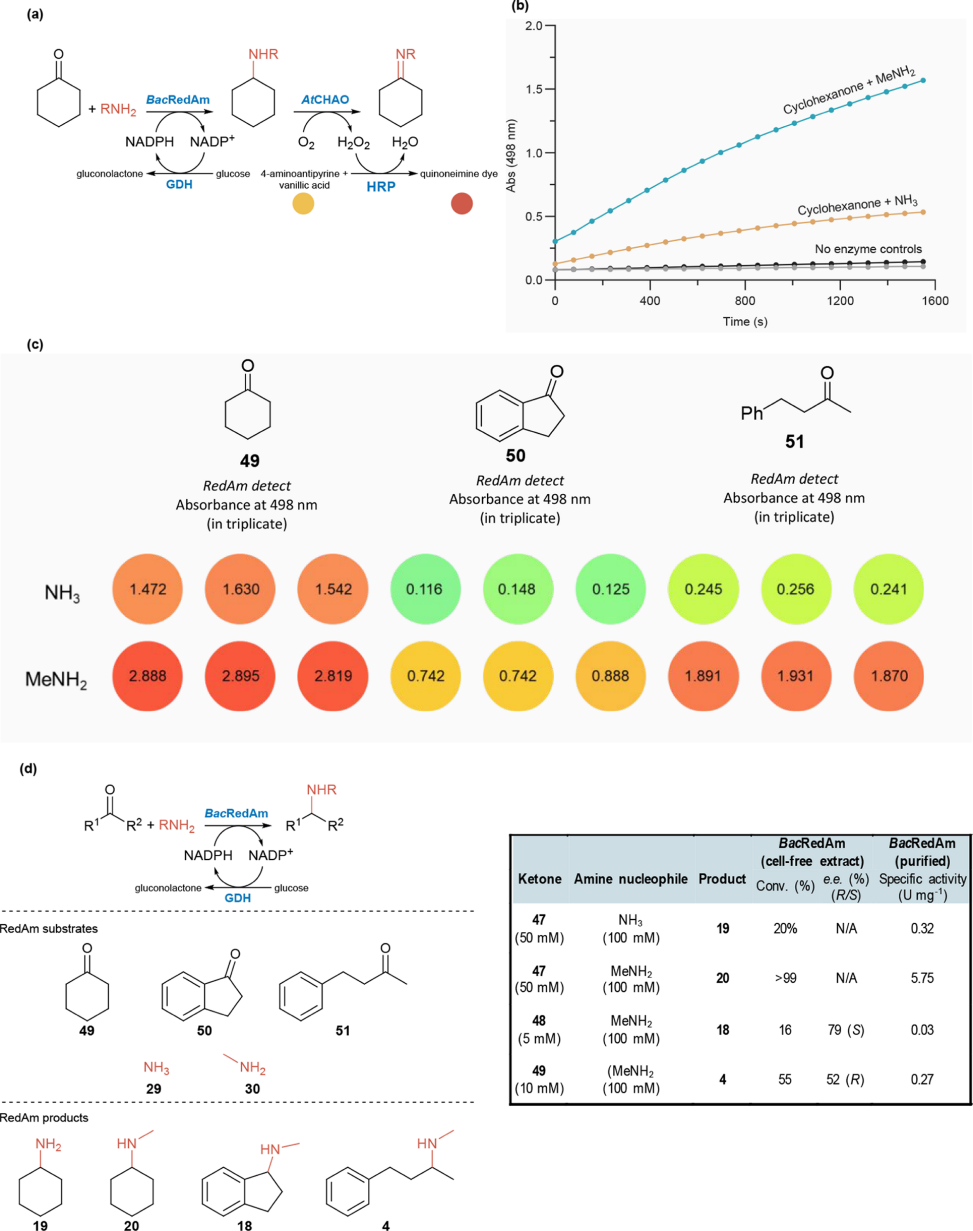
Validation of the *RedAm detect* assay for colorimetric/spectrophotometric monitoring of reductive aminase activity in cell-free lysates. (a) An overview of the *RedAm detect* reaction scheme, incorporating glucose dehydrogenase-based NADPH recycling system. (b) Kinetic tracking of reductive aminase activity of *Bac*RedAm-catalyzed reductive amination of cyclohexanone with methylamine or ammonia using the *RedAm detect* assay. (c) *RedAm detect* assay implemented as end point photometric quantification of RedAm-catalyzed reductive amination. Reaction monitored through absorbance measurement at 498 nm; performed in triplicate. (d) Corresponding GC-MS/HPLC data showing conversion, *e.e.*, and specific activity values. Level of activity observed with the *RedAm detect* assay correlates with conversion and specific activity values, validating the assay performance. Biotransformation reaction conditions: ketone (5–50 mM), methylamine/NH_3_ (100 mM), 20–100 mM glucose, 0.5 mM NADP^+^, 50 μL *Bac*RedAm as fresh lysate prep, 0.3 mg mL^–1^ glucose dehydrogenase (GDH, lyophilized cell-free extract), in 0.5 mL 100 mM Tris-HCl (pH 8.5). The reaction mixtures were incubated at 30 °C for 24 h, and conversion rate to product were determined by GC-MS or HPLC analysis.

Subsequently, we explored the feasibility of kinetically tracking the aminase reaction by simultaneously adding buffers A1 and B1 (both prepared in 100 mM Tris-HCl buffer, pH 8) and monitoring the increase in absorbance over time, as shown in Figure 3. A significant time-dependent increase in absorbance was observed for the enzyme reaction compared with control reactions lacking either RedAm or the amine nucleophile (Figure 3b).

To improve the cost-effectiveness of our system for extensive screening of enzyme libraries, glucose dehydrogenase (GDH)-based recycling of NADPH was incorporated into our *RedAm detect* assay. Results obtained with buffer A2 (containing catalytic amounts of NADP^+^, glucose, and GDH) were comparable to those obtained with the NADPH-based buffer A1. We then implemented *RedAm detect* as an end point assay to monitor the reductive amination of cyclohexanone **49**, 1-indanone **50**, and 4-phenyl-2-butanone **51** with ammonia and methylamine, Figure 3c.

GC-MS and HPLC analysis of biotransformation reactions confirmed the reductive amination of cyclohexanone **49** with ammonia and methylamine, as well as the amination of 1-indanone **50**, and 4-phenyl-2-butanone **51** with methylamine afforded the corresponding amine products, cyclohexylamine **19** (20% conv.), and *N*-methyl-cyclohexylamine **20** (>99% conv.), *N*-methyl-2,3-dihydro-1H-inden-1-amine **18** (12% conv., 79% *e.e*., (*S*)) and *N*-methyl-4-phenylbutan-2-amine **4** (55% conv., 52% *e.e*. (*R*)), respectively, Figure 3d. These conversion values and the specific activity for these reactions, determined with purified *Bac*RedAm, correlate well with the activity measured using the *RedAm detect* assay (Figure 3). The imperfect stereoselectivity of *At*CHAO allows us to monitor the amination of prochiral ketones **50** and **51** using the *RedAm detect* end point assay irrespective of the stereoselectivity of the investigated RedAms. However, a longer incubation time is required to allow CHAO to oxidize the slow-reacting (*R*)-amine enantiomer. We propose that combining an (*S*)-selective amine oxidase (e.g., MAON-D9/CHAO) with an (*R*)-selective amine oxidase (e.g., an engineered HDNO variant^20^ or an engineered pkDAO variant)^21^ may improve the sensitivity of *RedAm detect* in monitoring the amination of prochiral carbonyl compounds. In addition, several amine oxidases have been developed^22^ including those that accept bulkier amine substrates (e.g., MAON-D11,^18^ CHAO PT.1 ^23^) and variants that tolerate bulkier *N*-substituents (e.g., MAON-D5, *Bo*CHAO Y321I/M226T).^22,24^ This rich toolbox of amine oxidases makes it likely that a suitable amine oxidase partner will be found through a literature search for *RedAm detect*-based screening of various synthetic targets.

### *RedAm Detect* Reveals Novel Activity of *Msme*AmDH Toward Reductive Amination with Methylamine

To investigate the scope of applicability of *RedAm detect* in reductive amination reactions catalyzed by aminases other than RedAms, such as amine dehydrogenases (AmDHs), we examined *RedAm detect* performance by screening three AmDHs: *Msme*AmDH,^25^ engineered *Lf*AmDH-M_0_,^26^ and chimeric *Chi*AmDH,^27^ toward the reductive amination of cyclohexanone **49** with ammonia and methylamine, Table 1. To simulate reaction conditions typically used in the reductive amination with AmDHs, such as high concentrations of ammonia, we tested and confirmed the retention of activity of the downstream *At*CHAO-HRP system in 0.5–1 M ammonium chloride/100 mM Tris buffer (pH 8.0). The *RedAm detect*-based screening of AmDH activity in the reductive amination direction was then implemented as an end point assay. AmDH-mediated amination was run initially using buffer A3 (containing catalytic amounts of NAD^+^, glucose, GDH, ketone, ammonium chloride, or methylamine, in 100 mM Tris-HCl, pH 8.5) and incubated for 18 h, after which buffer B1 (containing all components to drive the amine oxidase and the downstream HRP reactions) was added to the reaction.

**Table 1:**
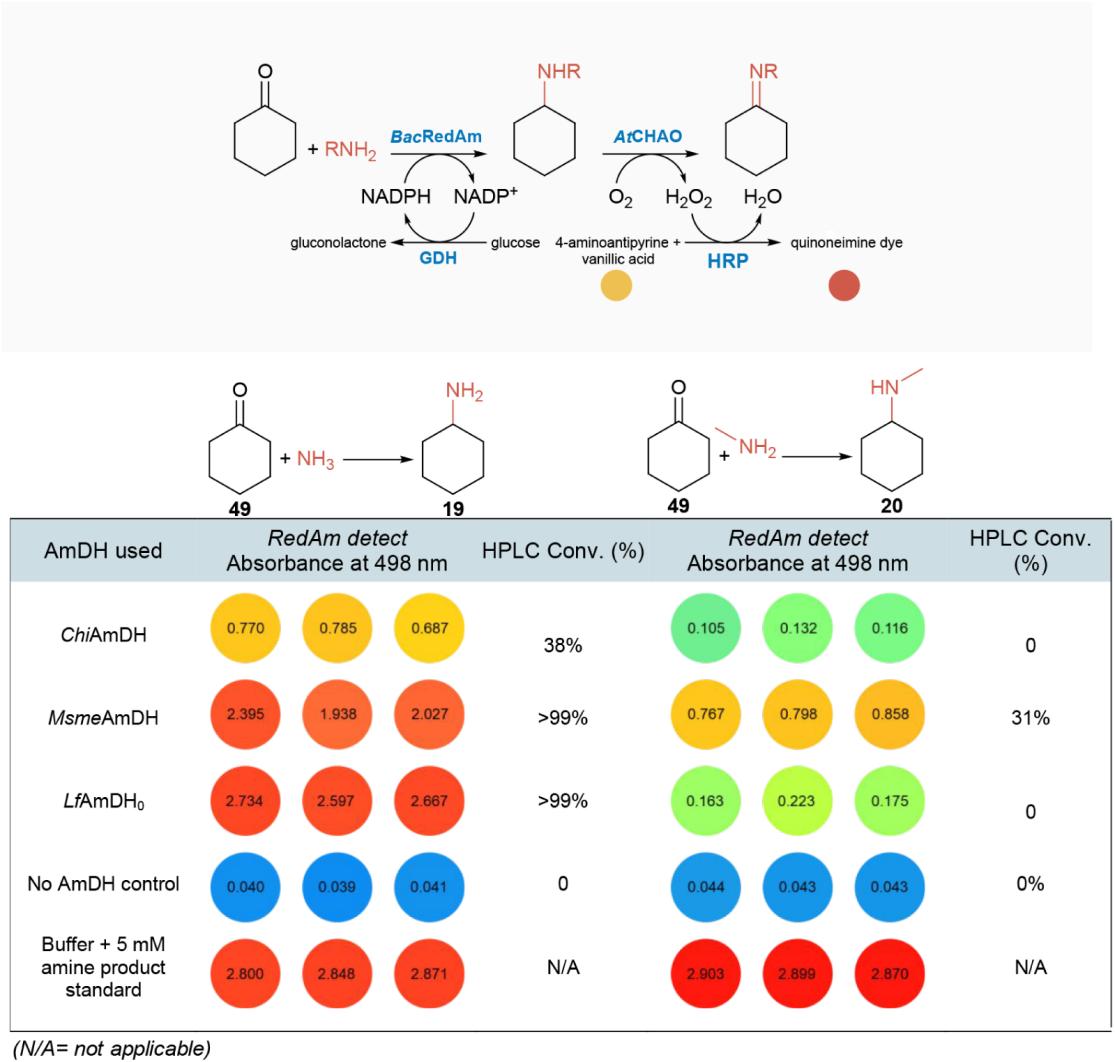
Monitoring Reductive Aminase Activity of Amine Dehydrogenases (*Am*DHs) Using *RedAm Detect* ^a^

a Increase in absorbance at 498 nm indicates product formation.

Colorimetric inspection and photometric measurements from the *RedAm detect*-enabled monitoring of AmDH-mediated biotransformation reactions showed that all three AmDHs investigated (*Chi*AmDH, *Msme*AmDH, *Lf*AmDH_0_) catalyzed the amination of cyclohexanone with ammonia, with both *Msme*AmDH and *Lf*AmDH displaying superior activity compared to *Chi*AmDH, Table 1. In contrast, only *Msme*AmDH was capable of catalyzing the reductive amination of cyclohexanone with methylamine to form the *N*-methylamine product **20**, Table 1. HPLC conversion data correlate with the activity observed with *RedAm detect* and confirm the ability of *Msme*AmDH to couple methylamine with cyclohexanone (31% conv.), a rare RedAm activity for *Am*DHs, which has previously not been reported for this enzyme, Table 1.

### *N*-Alkyl-*L*-Amino Acid Dehydrogenase *Dp*kA Capable of Using Ammonia as a Nucleophile

Our investigation then shifted to assessing the performance of the *RedAm detect* system in the reductive amination catalyzed by *N*-alkyl-*L*-amino acid dehydrogenases, an emerging enzyme class capable of catalyzing the reductive amination of α-ketoacids/ketoesters with methylamine and other alkylamines.^11,12,28^ We employed *N*-methyl-*L*-amino acid dehydrogenase from *Pseudomonas putida* (*Pp*DpkA),^12^ which has been reported to catalyze the reductive amination of α-keto acids/esters with methylamine and other alkylamines but is inert toward amination with ammonia.^12^ Given the shared kinetic and catalytic mechanisms between RedAms and DpkAs, despite being nonhomologous by primary sequence, we were particularly interested in determining whether DpkAs could also catalyze the amination with ammonia, akin to RedAms.

To develop a fit-for-purpose *RedAm detect* assay for monitoring the reductive amination of keto-acids/esters, we identified a commercially available *L*-amino acid oxidase from *Crotalus atrox* (*Ca*LAAO) for the oxidation of the α-amino acid/ester formed during this reaction. A substrate profiling study was performed against a panel of 17 α-amino acids/esters **26**, **27**, and **52**–**66**, Figure 4a. *Ca*LAAO oxidized a broad range of these α-amino acids/esters but displayed a clear preference for more hydrophobic substrates, such as *L*-homophenylalanine **52**, *L*-leucine **59**, *L*-methionine **61**, *L*-phenylalanine **65**, and *L*-tyrosine **66**. Modest activity was observed for *L*-histidine **64**, *L*-valine **55**, *L*-arginine **63,** and methyl-*L*-phenylalaninate **26**, while only trace or no activity was observed with smaller and polar amino acids such as glycine **53**, alanine **54**, *L*-threonine **56**, *L*-serine **58**, and *N*-methyl-*L*-leucine **60**.

**Figure 4:**
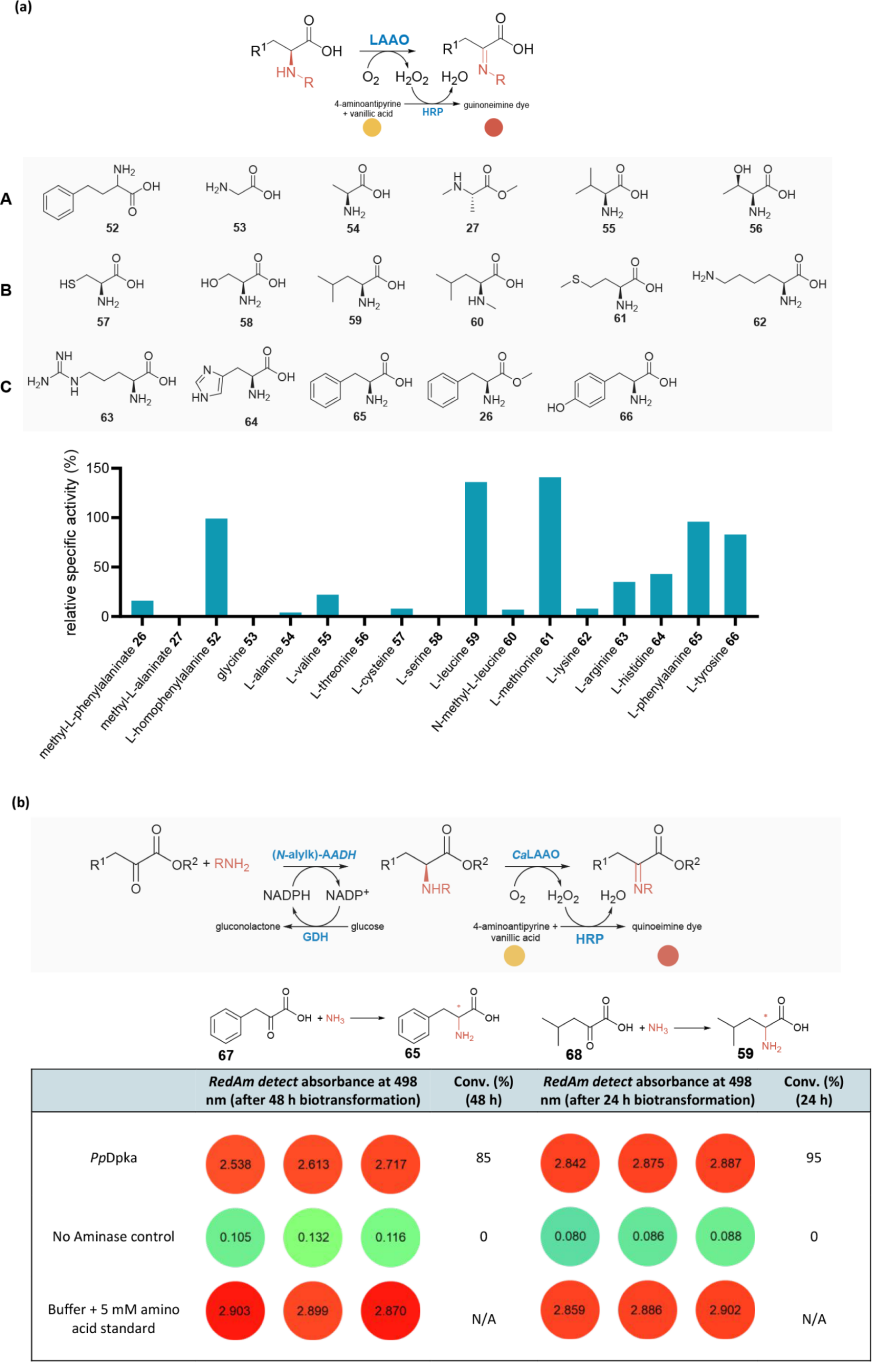
Measuring aminase activity in the reductive amination of α-keto acids with ammonia using *RedAm detect*. (a) Substrate profiling of *L*-amino acid oxidase from *Crotalus atrox* (*Ca*LAAO) against a panel of amino acids/esters. (b) *RedAm detect*-enabled monitoring of reductive amination of α-keto-acids with ammonia, using the DpkA-*Ca*LAAO-HRP *RedAm detect* set up. Buffer B2 was used for the substrate profiling of *Ca*LAAO. For *RedAm detect*, buffer A2 was used for the amination reaction step, incubated for 24–48 h, after which *Ca*LAAO/HRP-based buffer B2 was then added and further incubated overnight. Composition of buffers A2 and B2 can be found in Tables S1 and S2, Supporting Information. *Ca*LAAO, *L*-amino acid oxidase from *Crotalus atrox*.

Subsequently, the Dpka-mediated amination of α-keto acids **67** and **68** with ammonia toward the synthesis of *L*-phenylalanine **65** and *L*-leucine **59**, respectively, was monitored using the DkpA-*Ca*LAAO-HRP *RedAm detect* system. Here, *Ca*LAAO replaced CHAO/MAON as the amino acid oxidase, Figure 4b. Interestingly, the *RedAm detect* system revealed a novel DpkA activity, namely, the reductive amination of α-keto acids with ammonia to form both *L*-phenylalanine **65** and *L*-leucine **59** in high conversion of up to 95%.

This work demonstrates the potential of *RedAm detect* to uncover new enzymatic activities, highlighting its potential in enzyme discovery. Notably, the detection of *Msme*AmDH’s activity toward the reductive amination of cyclohexanone **49** with methylamine and *Pp*DpkA’s ability to catalyze the reductive amination of α-keto acids with ammonia underscores the broader applicability of RedAm detect in the discovery of novel amine-forming enzymes.

### Rapid Functional Mapping of Critical Residues in Reductive Aminases Enabled by *RedAm Detect*

We next sought to apply *RedAm detect* to rapidly scan for catalytically critical residues in RedAms. Traditionally, essential catalytic residues in RedAms have been identified by gauging the impact of substituting putative essential residues with one or two other amino acids by using site-directed mutagenesis, typically focusing on alanine variants. For example, the essential catalytic residues D169, Y177, and N93 in *Asp*RedAm and other RedAms were identified through this approach.^3,29^ While site-saturation mutagenesis (SSM) is commonly used to optimize enzyme performance for synthetic applications, it is rarely utilized to investigate enzyme mechanisms or substrate specificity. The application of SSM in studying the roles of residues in RedAms remains unexplored. We propose that screening a site (partial) saturation library containing multiple distinct substitutions at a target position to determine the percentage of active clones/mutants and the pattern of mutations retaining wild-type activity could provide a clearer understanding of the specific roles of those target residues. For example, *Asp*RedAm’s D169, Y177, and N93, which are conserved as D188, Y196, and N113 in *BacRe*dam, and D173, Y181, and N97 in *Ma*RedAm^13^ (a fungal reductive aminase from the Antarctica-inhabiting fungus *Mortierella antarctica*), respectively, could be studied in this manner.

Using the reductive amination of cyclohexanone **49** with methylamine **30** as the prototype reaction, we screened the libraries at these positions as lysate preparations using our *RedAm detect* assay to identify positions critical to RedAm activity. RedAm variants exhibiting >0.5 fold of the wild-type activity under the same reaction conditions were considered to have retained wild-type activity. This activity threshold was chosen after carefully reviewing the range of activity exhibited by different RedAms for this substrate combination reported in the literature and our comparative screening of RedAms available in our lab toward this substrate combination. By applying this cutoff criterion, we determine the percentage of clones retaining >0.5 fold of wild-type activity. Investigating the role of catalytic residues by screening site saturation libraries in this manner allowed us to confirm the critical (essential) roles that have been proposed for D188, Y196, and N113 in RedAm catalysis as <10% of mutants from these libraries retained comparable levels of activity to the wild type demonstrated in both *Bac*RedAm (D188, Y196, and N113) and *Ma*RedAm (D173, Y181, and N97), Figure 5.

**Figure 5:**
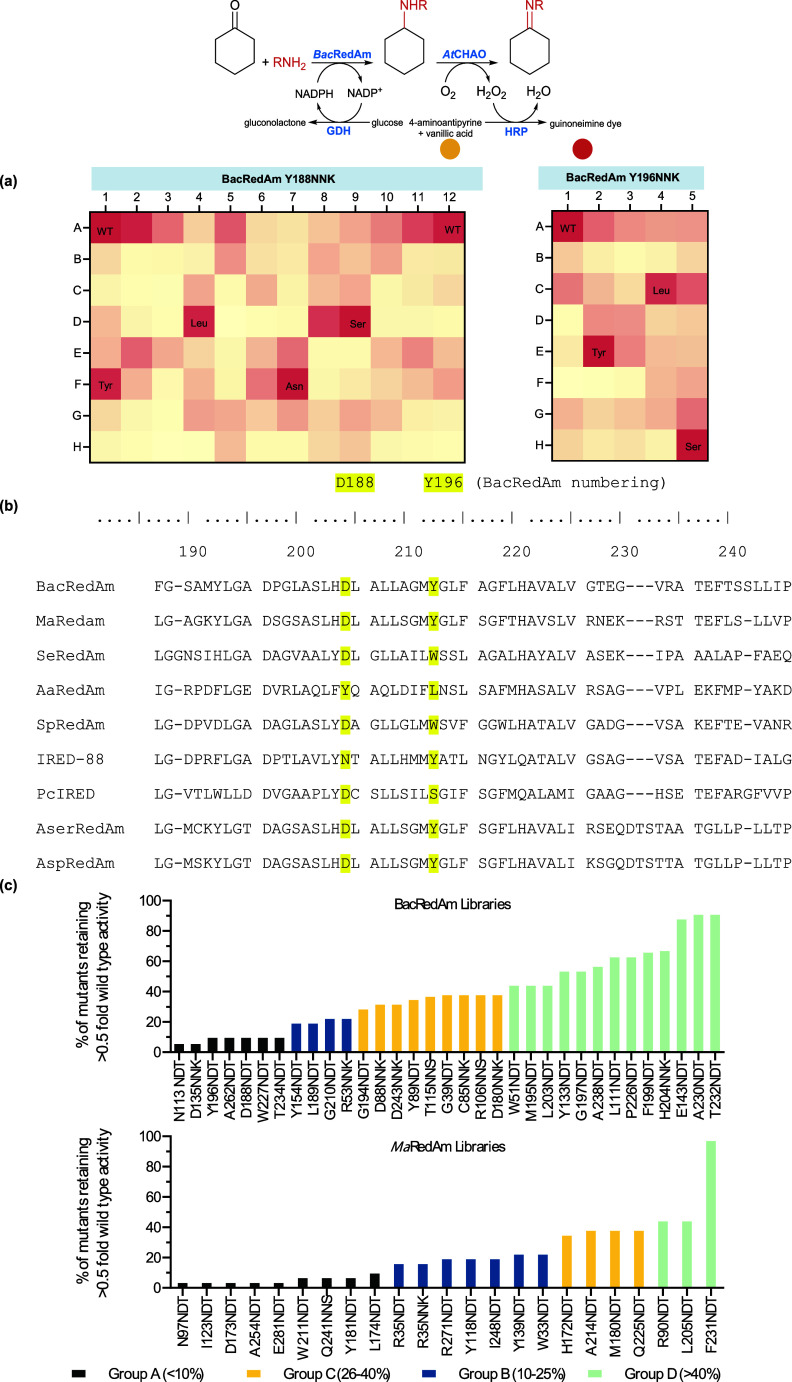
Gauging the tolerance of residues to combinatorial mutations as an indication of their critical roles. (a) Mutational scanning of two proposed essential catalytic residues in RedAms, D188 and Y196, from functional screening of site (partial) saturation libraries (NNK/NDT) at these positions, showing sequenced mutations of variants that retained activity comparable to that of the wild type for D188 and Y196. (b) Multiple sequence alignment of some characterized wild-type RedAms showing the precedence of the identified mutations in other wild-type RedAms. (c) Charts showing the degree of effect on RedAm activity for each combinatorial site (partial) saturation library of investigated residues in *Bac*RedAm and *Ma*RedAm, respectively.

We further examined the pattern of substitutions observed in mutants that retained activity at the D188 and Y196 positions in *Bac*RedAm (Figure 5a). These residues are proposed to play essential roles in RedAm catalysis by mediating substrate anchoring and (de)­protonation of the substrates and intermediates before and after nucleophilic attack.^29–32^ Their proposed roles in the RedAm’s acid–base catalytic mechanism would indicate that amino acids with protic side chains occupy these positions. Functional analysis of 96 mutants from the D188NNK library and Sanger sequencing of mutants retaining comparable activity with the wild-type *Bac*RedAm identified D188L, D188S, D188N, and D188Y mutations, as shown in Figure 5a. Similarly, Sanger sequencing of three active mutants from the functional screening of 40 mutants from the Y196NDT library identified one silent mutation, while the other two active mutants carried Ser (Y196S) and Leu (Y196L) substitutions, as shown in Figure 5a.

Intrigued by the presence of amino acid residues bearing aprotic side chains (e.g., Leu and Asn substitutions), we performed multiple sequence alignments of various characterized RedAms and confirmed that such substitutions are present in some wild-type homologues. For example, the recently characterized reductive aminases, IR007 (AaRedAm)^5^ and IRED-88 ^6^ feature Tyr (Y165) and Asn (N178) at the D188 equiv position (*Bac*RedAm numbering), respectively, whereas Leu (L173), Ser (S181), and Trp (W177) are found at the Y196 position (*Bac*RedAm numbering) in IR007 (AaRedAm),^5^ PcIRED,^33^ and *Sp*RedAm,^4^ respectively. We further examined the consensus pattern at the D188 and Y196 positions (*Bac*RedAm numbering) in >1,300 homologues. The D188 position is occupied predominantly by Tyr (47%), Asp (41%), Glu (8%), and Asn (3%), while the Y196 position is predominantly occupied by Trp (38%), Tyr (24%), Leu (20%), and His (15%).

These analyses reveal that, at least for critically important residues in RedAms, nature has primarily explored the potential mutability and/or variation of residues, as evidenced by a similar pattern of variations and/or substitutions at the D188 and Y196 positions in wild-type homologues versus active mutants from D188 and Y196 libraries retrieved from our screening. This suggests that for the key functional residues identified in this study, and by extension in the RedAm family, exploring residue variations/substitutions that nature has already selected in multiple homologues at the target positions can provide a straightforward, semirational engineering of these enzymes for application. This, for example, can be implemented through reconstructing ancestral sequences^34–36^ or by simply substituting the wild-type residue with other naturally occurring alternative residues at the equivalent positions in other homologues.

We then aimed to extensively investigate several conserved residues in *Bac*RedAm and a fungal reductive aminase from the Antarctica-inhabiting fungus *Mortierella antarctica* (*Ma*RedAm),^13^ irrespective of whether they are situated in the active site. Using multiple sequence alignment, residues with protic or bulky side chains and with high sequence conservation (>80% of the same or similar residues) were identified, with neighboring residues occasionally included. Site (partial) saturation libraries (NDT/NNK/S) with sufficient mutational variability, as confirmed by Sanger sequencing, were successfully constructed at 33 distinct positions in *Bac*RedAm and 22 unique positions in *Ma*RedAm, Figure S1. Again, we determined the percentage of clones that retained ≥0.5 fold wild-type activity in each library. This demonstrated that the positions screened have some impact on the overall enzyme efficiency, yet their tolerance to substitutions with alternative residues varied significantly, as shown in Figure 5c. In total, 56 single-site (partial) saturation libraries (NDT or NNK/S) of *Bac*RedAm and *Ma*RedAm were successfully screened using our *RedAm detect* assay toward the reductive amination of cyclohexanone **49** and methylamine **30**, Figure 5c. Analyzing data generated from our high-throughput screening of multiple positions using *RedAm detect* enabled classification of residues into four groups, **A**–**D,** depending on the degree of tolerance to alternative mutations with other amino acids (Figure 5).

Group A consists of positions where fewer than 10% of clones from their NNK/NDT libraries retained wild-type activity, indicating that these residues are highly critical for RedAm function/substrate specificity and are least tolerant to mutations to other amino acids. Additionally, residues where a substantial proportion of variants exhibited ≥1.3-fold activity improvement compared to the wild-type enzyme, such as F199 in *Bac*RedAm and F184 in *Ma*RedAm, were classified in Group A. Based on these criteria, 11 positions were categorized in Group A: N113, D135, D188, Y196, F199, W227, T234, Q257, A262, S270, and D297 in *Bac*RedAm, along with their equivalent residues in *Ma*RedAm (N97, H120, D173, Y181, F184, W211, Y218, Q241, A254, N246, and E281), Figure 5c, 6, and Table S4. Notably, D188, Y196, and N113 (and the equivalent residues D173, Y181, and N97 in *Ma*RedAm), which have been identified to act as essential catalytic residues in various RedAms, were also identified as critically important residues in this study, lending credence to our *RedAm detect* assay’s performance in pinpointing crucial residues.

**Figure 6:**
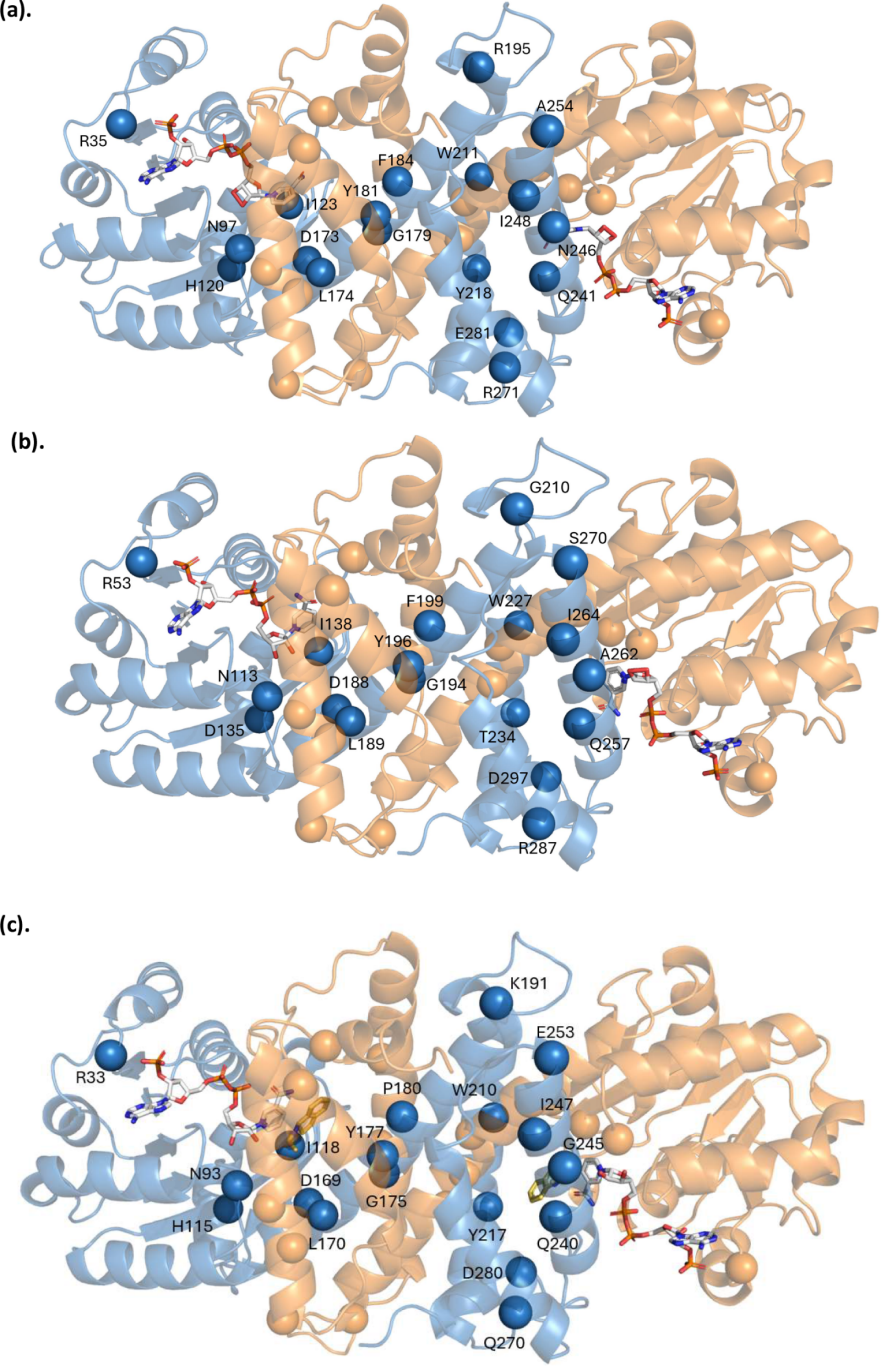
Dimer view of the ΑlphaFold model of *Ma*RedAm (a) and *Bac*RedAm (b) with docked-in cofactor (NADP­(H)), showing catalytically critical residues (as spheres) and dimer view of the well-characterized *Asp*RedAm complex (c) bound to NADP­(H) and the amine product rasagiline (PDB: 5G6S) and showing equivalent positions.

Nine positions, including R33, R35, Y118, Y139, L174, G179, R195, I248, and R271 (*Ma*RedAm numbering, equivalent positions in *Bac*Redam W51, R53, Y133, Y154, L189, G210, G194, I264, and R287, respectively), were also found to be critical for RedAm activity, as only 10–25% of variants at these positions retained wild-type activity levels and were assigned to Group B, Figure 5c, 6, andTable S4.

Group C comprised 11 positions: Y118, Y154, H172, A214, M180, Q225, R90, L205, D72, D165, L87, D227, and N73 in *Ma*RedAm, or their equivalent residues in *Bac*RedAm (H187, A230, M195, Q241, R106, S221, D88, D180, L103, D243, and Y89, respectively). These residues had moderate effects on activity, with up to 40% of variants maintaining activity comparable to that of the wild-type enzyme (Figure 5c and Table S4). The remaining ten residues exhibited relatively mild or neutral effects when mutated, with 40–91% of mutants from site-saturation libraries at these positions maintaining activity similar to the wild type. These were classified as Group D, Figure 5c and Table S4.

Consequently, we propose that the residues in Group A, and, to a large extent, those in Group B, play crucial roles in RedAm catalysis and/or substrate/cofactor specificity. A survey of RedAm engineering efforts supports these proposed roles in well-characterized RedAms, Table S5. For example, the identified catalytically important positions D188, Y196, and N113 (*Bac*RedAm numbering) correspond to the three catalytic residues in *Asp*RedAm and RedAm homologues.^3,29^ These positions and their immediate neighboring residues may also act as valuable engineering targets for improving catalytic efficiency, expanding substrate scope or enhancing stereoselectivity,^37^ as has been demonstrated in other reductive aminases for more challenging substrate sets, including *Sp*RedAm.^4^ GSKIR46,^7^ IR007,^5^ and Table S5. Similarly, substitutions at positions I138, G194, I264, D297, T234, W227, D135, Y154, Q257, G210, A262, and D297 (BacRedAm numbering), identified as important in RedAms in this work, have led to beneficial mutational effects for other substrate combinations in other RedAms, Table S5.^4,5,7^

### Identification of Residues for Engineering Substrate Specificity in RedAms

To investigate the roles of residues in substrate specificity, we employed *RedAm detect* as an end point assay and rescreened the 56 *Bac*RedAm and *Ma*RedAm site saturation libraries against three reductive amination reactions. These include the amination of 1-tetralone **69** with methylamine, the amination of 4-phenyl-2-butanone **51** with methylamine, and the amination of **51** with ammonia. The biotransformation for the RedAm-catalyzed reductive amination step was performed with buffer A2 (containing 7.5 mM ketone, GDH, NADP^+^ and glucose, and either 100 mM methylamine or 250 mM NH_4_Cl, pH 8.5). Following 18 or 48 h (for tetralone amination) of incubation to allow amine products to accumulate, reaction buffer B1 for the downstream CHAO-HRP steps was added and further incubated overnight, after which spectrophotometric measurements (498 nm) and colorimetric visual inspection were performed.

The reductive amination of 1-tetralone **69** with methylamine toward synthesizing *N*-methyl-1,2,3,4-tetrahydronaphthalen-1-amine **4**, a precursor of sertraline, is a particularly challenging transformation for RedAms. We identified several positions in *Bac*RedAm (F199, W227, L111, T234, Q257, R53, Y196, W51, and T115) and in *Ma*RedAm (N241, R35, I123, K39, L174, and L205) that yielded mutants with ≥1.3-FIOP, Figure 7a. Biotransformation reactions showed significant improvement in conversion values obtained with the best-performing mutants (up to 9% compared to 2% with the wild-type enzyme; Figure 7b), although activity for this transformation remains low.

**Figure 7:**
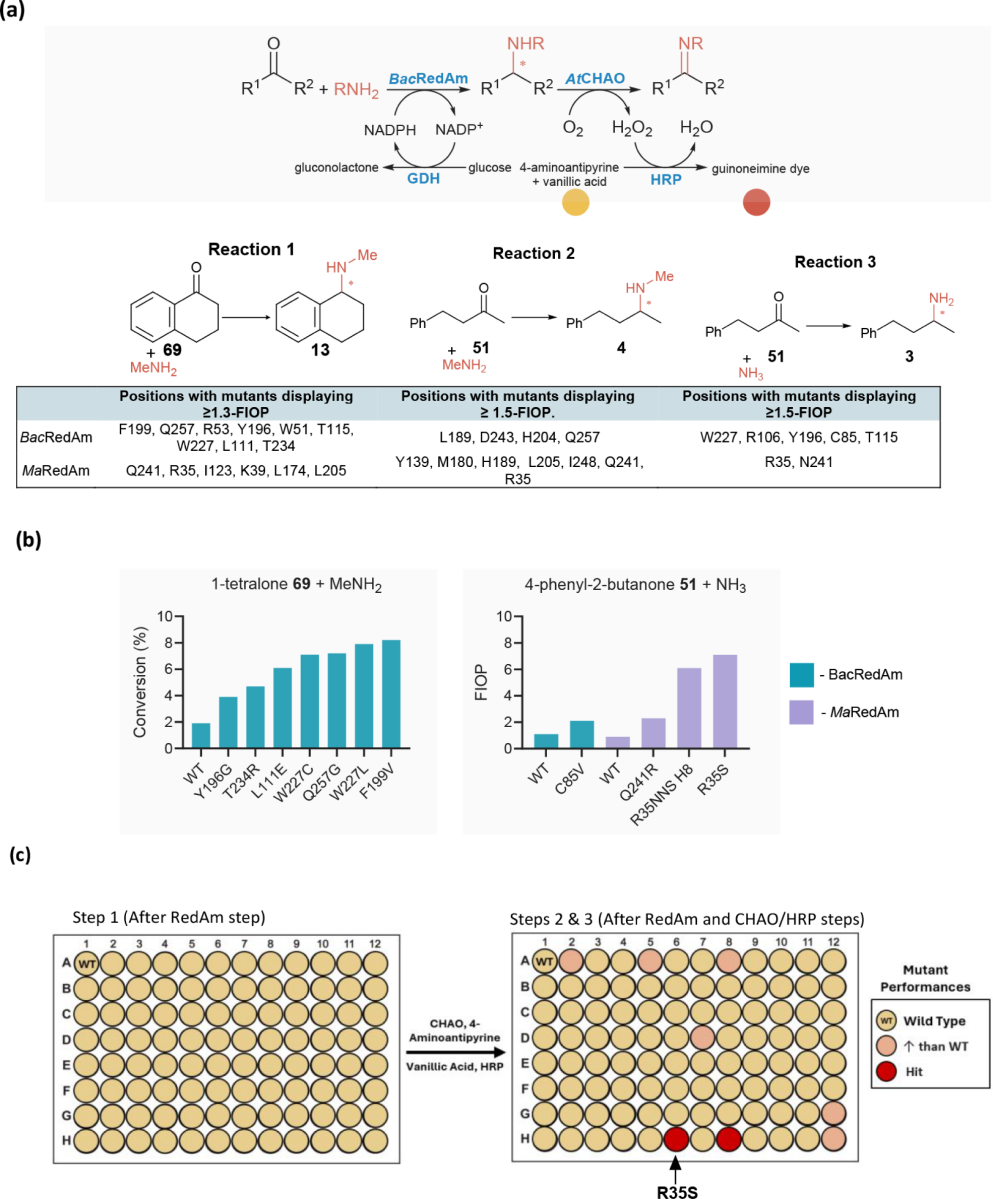
Screening of RedAm libraries across three reductive amination substrate combinations and identification of hotspot residues. (a) Positions in RedAms with mutants displaying significant improvement in activity compared to the parent enzyme. (b) Selected examples of variants displaying improved activity over that of the wild-type enzyme. (c) A sample plate of the *RedAm detect*-enabled screening of *Ma*RedAm R35NNS toward the amination of **51** with ammonia.

For the reductive amination of **51** with methylamine, four positions in *Bac*RedAm (L189, H204, D243, Q257) and six positions in *Ma*RedAm (Y139, M180, H189, L205, Q241, I248) yielded mutants displaying 1.5–7.2-FIOP. For the amination of 4-phenyl-2-butanone with ammonia, five positions from *Bac*RedAm saturation libraries at positions (C85, R106, Y196, H115, and Q257) and three positions in *Ma*RedAm libraries at Y139, R35, and N241 contain hits displaying 1.5–7 FOIP. Position R35 in *Ma*RedAM produced two of the most active hits, showing 6 and 7.2 FOIP toward this reaction, with sequencing results revealing R35S as the most active mutant, Figure 7c. Although RedAms can utilize ammonia, the activity toward amination with ammonia is generally low. The position highlighted in this study can serve as a hotspot for improving RedAms’ efficiency toward the reductive amination with ammonia.

Altogether, data from the four reactions (reactions 1–3, Figure 7, and cyclohexanone + methylamine, Figure 5) screened against the 56 RedAm libraries reveal positions R53, T115, Y154, L189, M195, Y196, W227, and Q257 in *Bac*RedAm or the equivalent positions in *Ma*RedAm (R35, T99, Y139, L174, M180, Y181, W211, and Q241, respectively) to have significant effects in at least three of the four reactions investigated. These positions may act as “universal” residues for engineering substrate specificity in these enzymes across different substrate combinations. Given that our screening did not cover the whole sequence length of these RedAms, the positions identified here are not an exhaustive list of residues that may act as targets for optimizing activity across several substrates. However, our approach to screening RedAms libraries across multiple substrate combinations should provide a template for identifying more residues that can be exploited as “universal” handles for engineering members of the RedAm family for a target synthetic application.

## Conclusion

In summary, we have developed a versatile *RedAm detect* assay for monitoring aminase activity in cell extracts, suitable for application in enzyme discovery and directed evolution of amine-forming enzymes. We demonstrated the broad applicability of *RedAm detect* assay in monitoring reductive amination of ketones with ammonia and methylamine, as well as the reductive amination of α-keto acids with ammonia, across three distinct enzyme families: RedAms, AmDHs, and AADHs. *RedAm detect* allowed us to uncover novel activity of *Msme*AmDH for the reductive amination of cyclohexanone with methylamine and DpkA-catalyzed amination of α-keto acids with ammonia.

Our *RedAm detect* assay is both timely and versatile. It can be applied for high-throughput screening of aminase activity in cell extracts for enzyme discovery and enzyme engineering projects, and it is amenable for use in multiple amine-forming enzyme families such as IREDs/RedAms, amine dehydrogenases,^25,38^ and *(N*-alkyl) amino acid dehydrogenases.^28,39^ Our system allows for the screening of reductive amination with ammonia and small alkylamines. By focusing on product formation rather than NADPH depletion, this method minimizes false positive detection, a common problem in NADP­(H) depletion, lysate-based screening systems. This assay presents significant potential for high-throughput screening and identification of amine-forming enzymes in functional metagenomics and directed evolution studies, offering a valuable tool for enzyme discovery and engineering.

Employing *RedAm detect*, we rapidly screened 56 site saturation RedAms’ libraries across four reductive amination substrate combinations. We identified R53, T115, Y154, W227, Q257, L189, and Y196 in *Bac*RedAm or the equivalent positions in *Ma*RedAm (R35, T99, Y139, W211, Q241, L174, and Y181, respectively) that may act as “universal” hotspots which can be used to optimize activity across different substrates. This work lays an important foundation for making RedAm engineering predictable, faster, and cost-effective.

## Supplementary Material



## Notes

Extended data, detailed experimental procedures, chiral HPLC chromatograms for product analysis, and sequence data (PDF)
