# Supplementary material for: Functional Mapping of Key Residues in Reductive Aminases Enabled by a High-Throughput RedAm Detect Assay
Source: JACS Au. 2025 Jun 27;5(7):3468–82. doi: 10.1021/jacsau.5c00512 (PMC12308443; doi:10.1021/jacsau.5c00512)
Supplement: Supplementary file 1 [file au5c00512_si_001.pdf]

Supporting Information for

Functional Mapping of Key Residues in Reductive Aminases Enabled by a High-throughput *RedAm Detect* Assay.

Jinming Xing, ‡ Georgie Orderley,‡ Ruth T. Bradshaw Allen, Nabieha N. Ahmad, Camille Gourjault, Ardil Akgul, Sephia O. Alhassan, Nichapa Ngernanek, Siddhika Salke, Godwin A. Aleku*

Institute of Pharmaceutical Science, Franklin-Wilkins Building, King's College London, 150 Stamford Street, London SE1 9NH, United Kingdom.
+ These authors contributed equally. * Correspondence: godwin.aleku@kcl.ac.uk

Section S1. Experimental Procedures

Chemicals and molecular biology reagents. Commercially available chemicals, inorganic salts, HPLC solvents, and reagents of high purity were purchased from Merck Life Science UK (Sigma-Aldrich, Dorset, UK), Fluorochem (Hadfield, UK), Thermo Fisher Scientific (Horsham, UK), and Acros Organics (Loughborough, UK). D-(+) glucose was also acquired from Merck Life Science UK (Sigma-Aldrich, Dorset, UK). Enzyme nicotinamide cofactors $NAD(P)^+$ and NAD(P)H were purchased from Cambridge Bioscience (Cambridge, UK). Media were purchased from Formedium (Hunstanton, UK).

The gene sequences of *Bac*RedAm, *Ma*RedAm, *Pp*DpkA and *At*CHAO were codon-optimised for expression in *E. coli*, synthesised by Twist Biosciences (South San Francisco, USA), and cloned into a pET28a (+) vector. The plasmid for MAON-D9 was kindly offered to us from Nick Turner's lab (Manchester, UK) and was produced as previously reported.[1] DNA primers were synthesised by Sigma-Aldrich (Dorset, UK). Chemically competent high-efficiency and subcloning efficiency *E. coli* cells (DH5α and BL21 DE3) were purchased from New England Biolabs (Hitchin, UK), as were the Q5 High-Fidelity PCR master mix, T4 Polynucleotide Kinase (PNK), T4 DNA ligase, and Dpn1. Fast Digest Dpn1 was obtained from Thermo Fisher Scientific (Horsham, UK). All gels, buffers, stains, and markers were also supplied from Thermo Fisher Scientific (Horsham, UK), as was the GeneJET plasmid miniprep kit for DNA and SDS-PAGE electrophoresis procedures. Lysozyme from chicken egg white, benzonase nuclease, and peroxidase from horseradish (P8250-5KU) were sourced from Merck Life Science UK (Sigma-Aldrich, Dorset, UK), while GDH 101 was sourced from Johnson Matthey (Cambridge, UK), and lyophilised CFE AmDHs were sourced from Prozomix (Haltwhistle, UK). L-amino acid oxidase from *Crotalus atrox (*CaLAAO*)* was purchased from Merck (Sigma-Aldrich, Gillingham, United Kingdom).

Primer design, mutagenesis, and library validation. The Protein sequences of *Ma*RedAm and *Bac*RedAm were used as query sequences, and utilising protein BLAST, 400 homologues were retrieved from non-redundant protein databases available on NCBI. Using the UGENE bioinformatic tool, multiple sequence alignment was performed with MUSCLE to identify conserved bulky and protic residues in the RedAms homologue sequences. Residues with high conservation scores (>80%) were selected as targets for site-saturation mutagenesis (SSM).

PCR Primers for mutagenesis were designed using NEbaseChanger and synthesised by Sigma-Aldrich. Site-directed mutagenesis was performed according to the NEB Q5® mutagenesis protocol using the wild-type RedAm plasmid as a template. 3 µl of PCR amplification products were analysed by agarose gel electrophoresis, while the remainder were stored at 4 °C for ligation. Samples showing a band at around ~ 6kb (pET28a vector + RedAm insert) following agarose gel analysis were progressed for ligation. One-pot kinase-mediated phosphorylation, ligase-catalysed ligation, and Dpn1-catalysed

digestion (KLD reaction) were performed with a KLD (Kinase, Ligase, Dpn1) cocktail prepared in-house. KLD enzyme mix was made from 33.5µL T4 polynucleotide kinase (NEB® M0201S), 2µL T4 DNA ligase (NEB® M0202S), and 16.5µL of DpnI (NEB® R0176S). The recipe was adapted from DeMott et. al.[2] KLD buffer was made from 29µL ligase buffer and 71µL of deionised water. 4 µL of PCR product, 2 µL of KLD enzyme mix, and 4 µL of KLD buffer were mixed, and this ligation mixture was incubated in an Eppendorf thermocycler at 24 °C for 2h, followed by 37 °C for 1h, and used immediately for transformation or stored 4°C overnight.

Chemically competent *E.coli* DH5α cells were transformed with the KLD reaction product. To confirm that the target mutations have occurred and are evenly distributed, 4-6 colonies were randomly picked from each library/plate, and plasmid preps (using GeneJET plasmid miniprep kit) isolated from 10-15 ml overnight cultures were sequenced (Sanger sequencing) and analysed. Plates containing successfully mutagenised and diverse mutations, as determined by Sanger sequencing results, were washed with LB broth into a 50 ml falcon tube. The plasmids were then isolated from this culture to form an SSM plasmid library, which was stored at -20°C until needed.

Library expression and preparation. Chemically competent *E. coli* BL21(DE3) cells were transformed with 2-5 µl of plasmid library, plated on a kanamycin-supplemented agar plate, and incubated overnight at 37 °C. To prepare overnight cultures in 96-deep well plates, a single colony was inoculated into each well containing 400 µL of LB broth supplemented with kanamycin (35 µg ml^{-1}), and plates were then incubated overnight at 37 °C.

For protein expression, 10µl from each well of the overnight culture was transferred into an equivalent position in another sterile 96 deep-well plate, prefilled with 800 µl of LB autoinduction media per well supplemented with kanamycin (35 µg ml^{-1}). To the remaining overnight culture in 96 deep-well plates, 400 µL of 60% glycerol was added per well plate and stored at -80 °C as the library glycerol stocks. The expression plate was incubated at 37 °C for an initial 4 h and subsequently at 25 °C for a further 44-48 h. Cells from the expression library were centrifuged (4,000 r.p.m, 4 °C, 30 mins) and lysed with lysozyme-based buffer (1 mg ml^{-1} lysozyme from chicken egg white, 0.3 mg ml^{-1} polymyxin B, 1 µl ml^{-1} benzonase, in 30 mM Tris-HCl pH 7.5, incubated at 20 °C for 2 h). The clarified supernatant was transferred to a 96-well microtiter plate and used for screening.

Biotransformation. Biotransformation reactions using cell-free extract or purified enzyme preparation were performed employing glucose dehydrogenase (GDH)/$NADP^+$ as a cofactor recycling system. For RedAm/AmDH/DpkA-catalysed reductive amination of investigated ketones/ketoacids with $MeNH_2$ or NH_3, 1 ml reaction mixture contained 10-50 mM cyclohexanone, 100 mM $MeNH_2$ or 200 mM NH_4Cl (1000 mM NH_4Cl for AmDH reactions), 15-30 mM D-glucose, 0.3 mg ml^{-1} GDH (lyophilised cell-free extract), 0.5 mM $NADP^+$ in Tris-HCl buffer (100 mM, pH 8.5) containing 1-2% (v/v) DMSO. Amine

nucleophiles were supplied from 2 M buffered amine nucleophile solution stocks, pH 8.5. The reaction was initiated with the addition of RedAm or AmDH. The reaction volume was 1 ml with Tris-HCl buffer (100 mM, pH 8.5). Reactions were incubated at 28 °C with shaking at 200 r.p.m. for 18 -48h.

For analysis, reactions were quenched by adding 50 µL of 5 M NaOH and extracted twice with 500 µL tert-butyl methyl ether. The organic fractions were combined, dried over anhydrous MgSO4, and analysed on a GC-MS or normal-phase HPLC. The AmDH reaction was analysed on reverse-phase HPLC following derivatisation with Marfey's reagents.

***RedAm detect* assay.** To implement *RedAm detect* assay supplying (stoichiometric amounts) NADPH for the RedAm step, two buffers were prepared, namely **buffer A1** (containing 10-25 mM cyclohexanone **1**, 40 -100 mM methylamine (or ammonia), and 2-6 mM NADPH, in 100 mM Tris-HCl buffer, pH 8.5) and **buffer B1** (4 mM 4-aminoantipyrine, 4 mM vanillic acid, 1 U ml^{-1} HRP, *At*CHAO/MAON-D9 0.6 mg ml^{-1}, in 100 mM Tris HCl buffer, pH 8). For *RedAm detect* assay incorporating a GDH-recycling of NADPH, buffer **A1** was replaced with buffer **A2**, which contained 10-25 mM cyclohexanone **1**, 40-100 mM methylamine (or ammonia), and 0.5 mM $NADP^+$, 15-30 mM D-glucose, 0.3 mg ml^{-1} lyophilised CFE GDH, all in 100 mM Tris HCl, pH 8.5. For *Am*DH reactions, 1 M ammonium NH_4Cl was used, using buffer A3, **Table S1**.

A microtitre plate was prefilled with 100 µl (per well) of reaction buffer A1 or A2. To this plate was added 20 µL of clarified RedAm lysate from the lysate library plate into the corresponding well in the reaction plate. A1/A2 reaction plates were incubated at 30 °C, 100 r.p.m shaking, for 10-20 min (for amination of cyclohexanone with methylamine reaction) or 30 min to 1 h (for amination of cyclohexanone with ammonia). Then, 100 µL of buffer B2 was added to each well and further incubated at 30 °C, 100 r.p.m shaking for 5-10 min. Absorbance change was monitored at 492/498 nm with an Envision multi-plate microtitre reader. For the AmDH-catalysed reductive amination of cyclohexanone with ammonia or methylamine, the reaction with buffer **A3** was incubated for 18-48h before adding buffer B1 and further incubated overnight. Similarly, RedAm catalysed the reductive amination of 4-phenyl-2-butanone, 1-indanone, and 1-tetralone, and DpkA catalysed the amination of ketoacids. The reaction with buffer A2 was incubated for 18-48h before adding buffer B1/2, which was further incubated overnight.

For the one-pot *RedAm detect* assay for kinetic tracking of reaction rate, buffers A1/A2 and buffer B were all prepared at pH 8. To a microtitre plate containing 100 µL of A1/A2 per well was added 20 µL of clarified RedAm lysate to the corresponding well; the reaction was incubated at 30 °C 100 r.p.m shaking for an initial 2 min, then buffer B was added, and automated measurements of absorbance change at 30-60 s intervals were performed at 492 or 498 nm using Perkin Elmer Envision microtitre reader. When necessary, a microplate stacker was used.

Method for making the models: MaRedAm and BacRedAm AA sequences were submitted to ColabFold[3] (Alphafold2 using MMseqs2, v1.5.5) to generate monomeric PDB models. NADPH was introduced to both models via Alphafill[4]. The transplant clash score (RMSd of the Van der Waals overlap between ligand and polymer atoms) for MaRedAm and BacRedAm were 0.08 and 0optimised transplant clash scores for both models were 0.08 Å, indicating minimal steric interference. Dimeric structures were generated by aligning the monomeric models with PDB 5G6S in PyMOL (version 2.5.7). Enzyme-cofactor models were subsequently optimised using YASARA (version 18.4.24) energy minimisation to ensure stable interactions between the enzyme and cofactor.[5]

Section S2. Extended Data

Table S1. RedAm detect: Reaction buffers for the aminase-catalysed step (step 1).

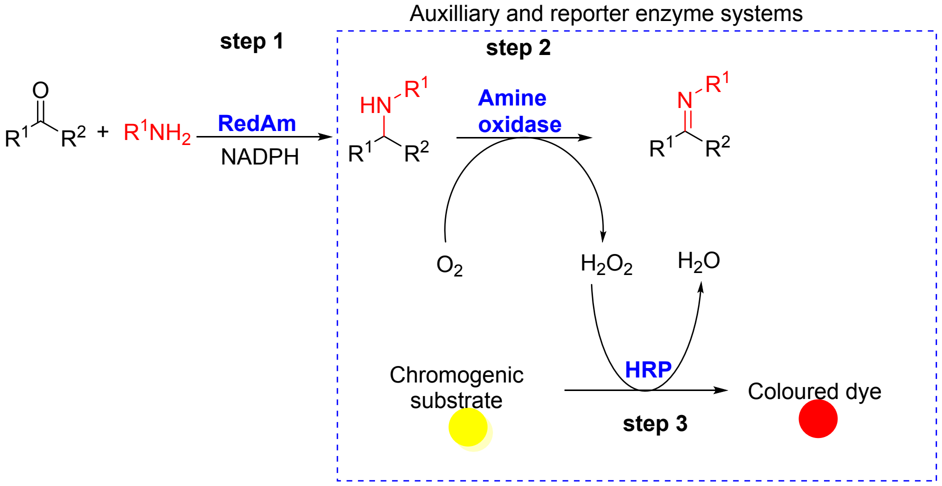

Measurement wavelength: 498nm/492nm		
Reaction buffer A1 for the Aminase-catalysed step (step 1) using stoichiometric concentrations of NAD(P)H. (Aminase: RedAms, DpKA, *Am*DH)		
**Buffer A1**	Ketone or ketoacid/ester	5 mM
	Amine nucleophile or ammonium chloride (from buffered stock solutions, pH 8.5).	50-250 mM
	NAD(P)H	2-6 mM
	DMSO	1-2%
	Buffer was prepared in 100 mM Tris-HCl buffer (pH 8.5).	
Reaction buffer A2 for the RedAm-catalysed step (step 1) incorporating Glucose dehydrogenase (GDH)-cofactor recycling. (Aminase: RedAms, DpKA, *Am*DH)		
**Buffer A2**	Ketone or ketoacid/ester	5-50 mM
	Amine nucleophile or ammonium chloride	50-250 mM
	Glucose	20-100 mM
	NAD(P)+	0.5 mM
	Glucose dehydrogenase (GDH) as lyophilised CFE	0.25 mg ml^{-1}
	DMSO	1-2%
	Buffer was prepared in 100 mM Tris-HCl buffer (pH 8.5).	

Reaction buffer A3 for the AmDH-catalysed step (step 1) incorporating Glucose dehydrogenase (GDH)-cofactor recycling.		
**Buffer A3**	Ketone or ketoacid/ester	10 mM
	Amine nucleophile or ammonium chloride	1000 mM
	Glucose	20 mM
	NAD(P)+	0.5 mM
	Glucose dehydrogenase (GDH) as lyophilised CFE	0.25 mg ml^{-1}
	DMSO	1-2%
	Buffer was prepared in 100 mM Tris-HCl buffer (pH 8.5).	

Table S2. Buffers for the Amine oxidase (or *L*-amino acid oxidase)/HRP-steps for substrate profiling and incorporation into the RedAm Detect system

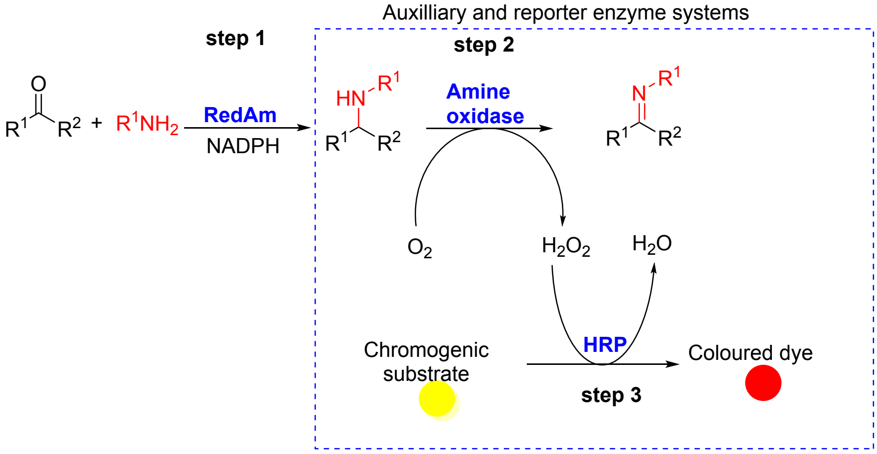

Measurement wavelength: 498nm/492nm

Reaction buffer **B1** for the amine oxidase/HRP reporter system, incorporating into the *RedAm detect* system		
**Buffer B1**	Amine substrate	0
	Amine oxidase (*At*CHAO/MAON-D9)	0.3-0.6 mg ml^{-1}
	4-aminoantipyrine	4 mM
	Vanillic acid	4 mM
	HRP	1 U ml^{-1}
	DMSO	1-2%
	The buffer was prepared in 100 mM Tris-HCl buffer (pH 7.5/8.0).	
Reaction buffer **B2** for the L-amino acid oxidase/HRP reporter system, incorporating into the *RedAm detect* system		
**Buffer B2**	Amine substate	0
	*Ca*LAAO	0.2 mg ml^{-1}
	4-aminoantipyrine	4 mM
	Vanillic acid	4 mM
	HRP	1 U ml^{-1}
	DMSO	1-2%
	The buffer was prepared in 100 mM Tris-HCl buffer (pH 7.5/8.0).	

Table S3. Spectrophotometric monitoring of Reductive amination reaction with RedAm detect assay.

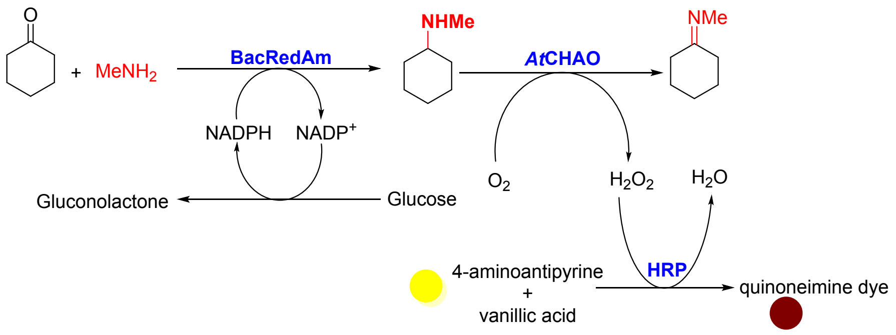

2 mins Incubation

	BacRedAm Y196NDT				BacRedAm F199NDT				BacRedAm W227NDT			
	1	2	3	4	5	6	7	8	9	10	11	12
**A**	0.11	0.11	0.19	0.11	0.20	0.34	0.12	0.22	0.12	0.19	0.30	0.12
**B**	0.15	0.12	0.19	0.13	0.22	0.12	0.12	0.17	0.12	0.11	0.19	0.15
**C**	0.12	0.11	0.15	0.11	0.12	0.18	0.12	0.19	0.19	0.11	0.27	0.26
**D**	0.12	0.11	0.16	0.31	0.13	0.13	0.12	0.28	0.13	0.11	0.14	0.16
**E**	0.11	0.12	0.21	0.14	0.11	0.12	0.11	0.12	0.19	0.12	0.15	0.13
**F**	0.15	0.11	0.22	0.11	0.40	0.15	0.12	0.14	0.18	0.13	0.12	0.14
**G**	0.11	0.11	0.22	0.13	0.24	0.19	0.34	0.30	0.13	0.31	0.14	0.19
**H**	0.12	0.14	0.25	0.12	0.12	0.15	0.12	0.15	0.17	0.12	0.11	0.11

Wells **A1**, **A5** and **A9** contained BacRedAm wild-type

30 mins Incubation

	1	2	3	4	5	6	7	8	9	10	11	12
**A**	0.65	0.40	0.49	0.41	0.79	0.51	0.74	0.82	0.72	0.55	0.65	0.41
**B**	0.33	0.21	0.23	0.23	0.97	0.60	0.69	0.78	0.42	0.29	0.25	0.23
**C**	0.42	0.23	0.28	0.49	0.75	0.81	0.51	0.65	0.34	0.25	0.30	0.39
**D**	0.19	0.41	0.27	0.26	0.61	0.78	0.40	0.83	0.35	0.27	0.27	0.24
**E**	0.25	0.73	0.42	0.32	0.31	0.59	0.70	0.35	0.35	0.25	0.35	0.23
**F**	0.19	0.17	0.27	0.26	0.88	0.40	0.51	0.72	0.42	0.32	0.28	0.24
**G**	0.18	0.18	0.27	0.41	0.83	0.81	0.86	0.88	0.42	0.37	0.36	0.37
**H**	0.42	0.27	0.49	0.26	0.54	0.82	0.76	0.77	0.69	0.44	0.38	0.29

Wells **A1**, **A5** and **A9** contained BacRedAm wild-type

60 mins Incubation

	1	2	3	4	5	6	7	8	9	10	11	12
**A**	1.04	0.70	0.80	0.70	1.26	0.81	1.17	1.29	1.15	0.89	0.98	0.69
**B**	0.46	0.31	0.28	0.35	1.52	0.97	1.14	1.25	0.69	0.49	0.43	0.36
**C**	0.74	0.37	0.40	0.85	1.24	1.31	0.89	1.05	0.52	0.42	0.46	0.61
**D**	0.28	0.71	0.38	0.39	1.01	1.27	0.69	1.28	0.58	0.47	0.45	0.37
**E**	0.40	1.19	0.63	0.52	0.50	0.94	1.15	0.59	0.50	0.42	0.54	0.34
**F**	0.28	0.27	0.37	0.42	1.42	0.67	0.89	1.15	0.66	0.53	0.46	0.36
**G**	0.25	0.29	0.35	0.68	1.33	1.28	1.39	1.36	0.68	0.61	0.61	0.58
**H**	0.71	0.45	0.79	0.41	0.88	1.36	1.25	1.25	1.12	0.74	0.64	0.48

Wells **A1**, **A5** and **A9** contained BacRedAm wild-type

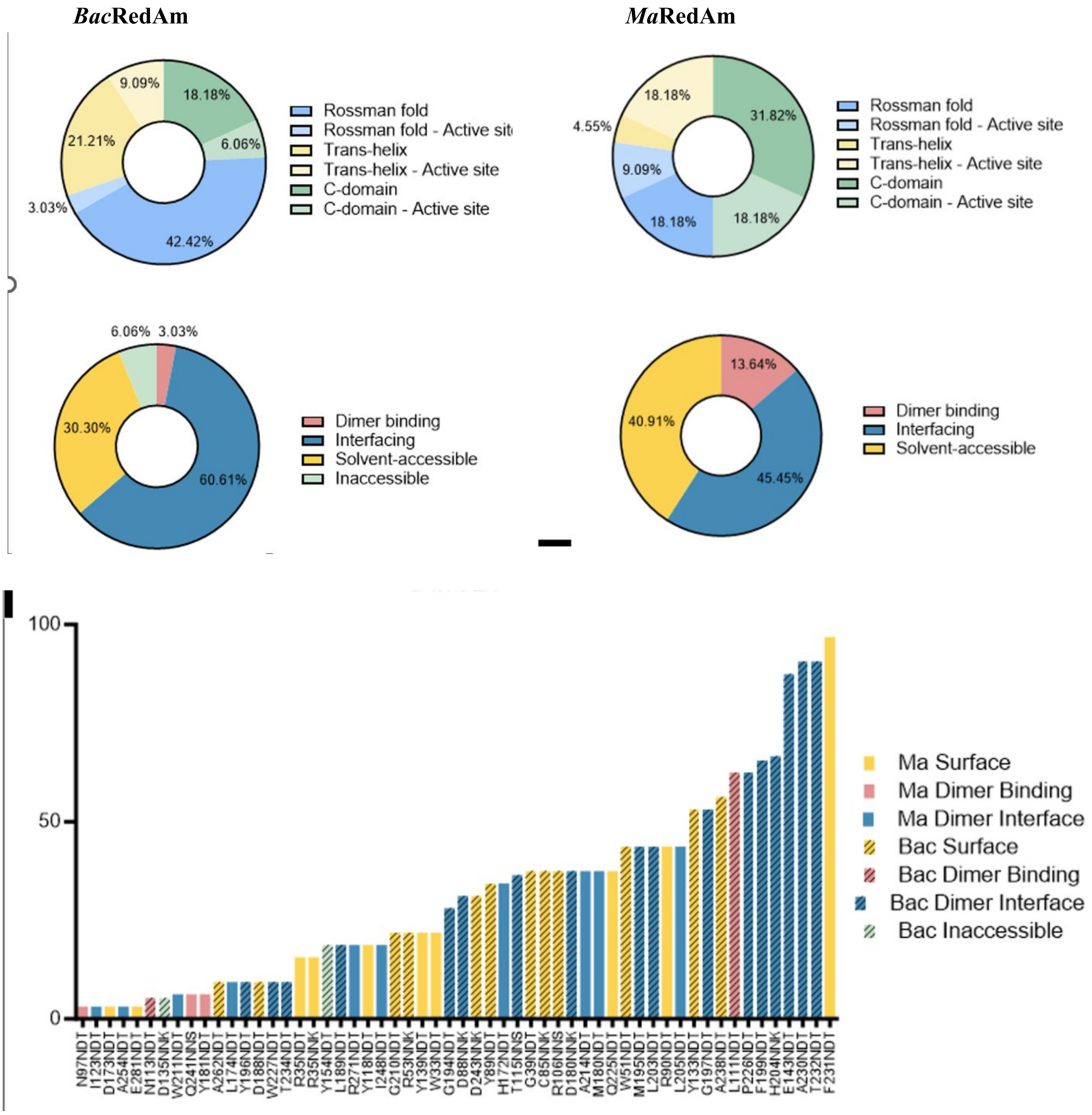

Figure S1. Analysis of the distribution of residues investigated in *Bac*RedAm and *Ma*RedAm,.

Table S4. Residues are categorised into groups A-D, depending on their critical roles in RedAm activity. *Ma*RedAm residues are presented, and equivalent residues in *Bac*RedAm are presented in parenthesis ().

Group A	Group B	Group C	Group D
<10% of variants from the site NNK/NDT library retain comparable activity to the wild type.	10-25% of variants from the site NNK/NDT library retain comparable activity to the wild type.	26-40% of variants from the site NNK/NDT library retain comparable activity to the wild type.	>40% of variants from the site NNK/NDT library retain comparable activity to the wild type.
N97 (N113)<br>H120 (D135)<br>I123 (I138)<br>D173 (D188)<br>Y181 (Y196)<br>W211 (W227)<br>Y218 (T234)<br>Q241 (Q257)<br>N246 (A262)<br>A254 (S270)<br>E281 (D297)<br>F184 (F199)	R35 (R53)<br>L174 (L189)<br>G179 (G194)<br>I248 (I264)<br>R195 (G210)<br>R271 (R287)<br>Y139 (Y154)<br>W33 (W51)<br>Y118 (Y133)	H172 (H187)<br>A214 (A230)<br>M180 (M195)<br>Q225 (Q241)<br>R90 (R106)<br>L205 (S221)<br>D72 (D88)<br>D165 (D180)<br>L87 (L103)<br>D227 (D243)<br>N73 (Y89)	F231 (Y247)<br>G182 (G197)<br>L222 (A238)<br>L95 (L111)<br>P210 (P226)<br>F184 (F199)<br>H189 (H204)<br>S128 (E143)<br>A214 (A230)<br>T216 (T232)

Table S5. This study identifies functionally important residues in reductive aminases, *Bac*RedAm, and MaRedAm. Equivalent positions in the well-characterised reductive aminase *Asp*RedAm and beneficial mutations at equivalent positions/adjacent positions in other RedAms are also presented.

(a). Functionally important residues identified in this study and precedence in other characterised RedAms. Proposed roles of these residues include acting as catalytic residues in the acid-base mechanism, contributing to RedAms' substrate and cofactor specificity/recognition.

Entry	*Ma*RedAm	*Bac*RedAm	*Asp*RedAm	Reported beneficial mutations in RedAms
1	R35	R53	R33	A60Q in IR46[6],
2	N97	N113	N93	T93 SpRedam[7]
3	H120	D135	H115	A112C in IR007[8]
4	I123	I138	I118	E120R/multiples in IR007[8], Y142S/R/V/T/Q in IR46[6], T122A in SpRedAm[7]
5	D173	D188	D169	D194 in IR46[6]
6	L174	L189	L170	A170C/M in SpRedAm[7]
7	Y181	Y196	Y177	L201Y/F in IR46[6], M176A in SpRedAm[7]
8	G179	G194	G175	L198M/C in IR46[6], L169T IR007[8]
9	F184	F199	F180	F180M in SpRedAm[7]
10	R195	G210	K191	R187K/H/Q/S/W in IR007[8]
11	W211	W227	W210	M206K/G/H/Q/R in IR007[8]
12	Y218	T2340	Y000217	0A213F/E/I/K in IR007[8], F214I/N in SpRedAm[7]
13	N246	A262	E245	D238G in IR007[8],
14	Q241	Q257	Q240	A264L, V265R/T and T260V/I/C in IR46[6],
15	I248	I264	I247	I240L/V in IR007,[8] A264L, V265R/T in IR46[6]
16	A254	S270	E253	
17	R271	R287	Q270	
18	E281	D297	D280	S273G in IR007[8]

Section S3. Gene sequences of enzymes characterised in this work, including *Ma*RedAm, *Bac*RedAm, *At*CHAO and *Pp*DpkA

>*Bac*RedAm
ATGAGGGAACCCATAGTAAGTGCTCACACAGAGCGCGCAGTCGAGTCTCGTGGCGCGGACCGTGGTTCTGCGGTT
ACCGTCATCGGCTTGGGTTCCATGGGTTCAGCCCTCGCCGGCGCGGTGCTGGAAGCGGGCTATCCGACGACCGTT
TGGAACCGCACGGCTGGTAAGGCAGAACCATTGGTGCGTAGAGGCGCGGCTCGCGCGGCGACGGTGGCGGAGGCG
GTGAGCGCGTCCCCGACCGTGATCGCCTGCGTGCTGGATTATCGTGCGTTACGTGAGATCCTGAGCACCGCGGGC
GACGCACTCGCTGGCCGTACCGTTGTTAATCTGACCAACGGTACACCGACCGAGGCCCGTGAAACCGCTGCTTGG
GTCGAGGGTCATGGTGCTCGTTACCTGGACGGCGGCATCATGGCAGTTCCGGAAATGATTGGTGGCGCGGAAAGC
CTTGTTCTGTATAGCGGTAGCGCCGAGGCGTTTGAAACCGTAGAGCCGGTTCTGCGTCGCTTCGGCAGCGCTATG
TACCTGGGTGCGGACCCGGGTTTGGCCTCGCTGCACGATCTGGCATTGCTGGCGGGGATGTACGGCCTGTTTGCA
GGCTTCCTGCACGCAGTGGCCTTAGTGGGTACGGAAGGTGTTCGTGCCACCGAGTTCACCAGCTCCCTGCTGATT
CCGTGGCTGCAGGCCATGACCGCGACTCTGCCTGAAGCTGCGGCGCAAATTGATGCAGGCGACTACGCAGCGACT
GGTTCTCGCCTGGACATGCAAGCGGTCGCGTTGGCGAACATTGTTGAGGCGAGCAGAAGCCAGGGTATCCGTCCG
GATCTTATGCTGCCGATTCAGGCATTGGTTGAGCGCCGTGTGGCAAAAGGTGGTGGCGGTGAAGATATCGCAGCG
GTGGTGGAGGAAGTTCGCGGATAA

>*Ma*RedAm
ATGACTTCTTCCTCCACTGTTAGCATTATCGGCCTTGGCGCCATGGGCTTGGCCCTTGCTGCCAAGTTTGTGGAG
AAGGGCTACACGACCACTGTTTGGAACAGATCCACCGAGAAGGCACTCAAGTTTGCTGCAGAGCACGAGAACGCG
CATGCCGCAACCACCGTGGCTCAAGGTCTAGAGGCTAGCAACCTGGTGATCATCTGTCTTCTCGACAACAAGGCC
GTTCGCGATACCATTGATCAAGCCCTTCCCTCCTTGGCGGGACGCATCGTTGTCAACCTGACTAACGGTACCCCT
GATGAGGGACGAGAGACTGGGGCCCTTGTGGCGGCTCAGGAAGGATCCAAGTACGTGCACGGCGGGATCATGGCA
ACTCCTTCGATGGTCGGCTCTCCGGCATCGGTGCTGCTGTACAGCGGATCTCTAGAAGCTTACACAGCGGTGGAG
AAGGATCTCGAGATCCTGGGTGCTGGCAAGTACCTCGGAGCCGACTCTGGATCGGCTTCGCTGCATGATCTGGCG
CTCCTGAGCGGGATGTATGGCCTCTTCTCTGGCTTTACGCATGCAGTGTCGCTGGTGCGGAACGAGAAGCGGTCG
ACCACGGAATTCCTGTCGCTTTTGGTGCCCTGGCTGACGGCGATGACGGGCTACCTGCACGTGCTGGGCAAGCAG
ATTGATGAGGGCGACTTCTCGTCTCTTGGGTCGAGTATTGAGATGCAGGTGCCTGCGATCAACAACATTGTGAAG
ACAAGCGAGGCACAGGGCGTGTCTGCGGATCTCATCCGGCCCATCCAAGGCTTGCTGGAGCGTGCGGTGGCGGTC
GGACGAGGTGGCGAGGAGATCTCGGCGCTGGTGGGCCTGAATGTGCTGGCGAGGAAGGCAGAGTAA

>*At*CHAO
ATGTGTCGAAGTAGACAAGCAGCTAGGTCAAAGCGCGAGGAGTCTGCTCTGACCCACCTAAACACCTATGAGAGC
GTGACCCCAGACCCGGATGTTGACGTTATTATCATCGGCGCGGGCATCAGCGGCTCCGCCGCGGCTAAGGCACTG
CATGACCAAGGTGCGAGTGTTCTGGTTGTTGAAGCGAACGATCGGATCGGTGGCAGAACGTGGACCGAGCAAGAG
GGTGCTCCGGGTGGACCGATTGATTACGGGGGTATGTTTATCGGCGAAACCCACACCCACTTGATTGAGCTCGGC
ACTTCCCTTGGCCTGGAAATGACCCCGAGCGGTAAGCCGGGTGATGACACCTACATCGTGGCAGGCAATGTGCTG
CGTGCCCCCGACGACCAGCTGGACCCTAACCTGCCGTTTGTTCCGGAATTTCTGTCTAGCCTGAAAGCGTTGGAC
GAATTGGCAGATTCAGTCGGTTGGGATCAGCCGTGGGCGTCGCCGAATGCGGCTGCGCTGGACAGCAAAACCGTG
GCCACGTGGCTGGCGGAGACGATTGAATCTGAGGAAGTTAGACGTCTGCATACTGTCATCGTTAATACGCTGCTG
GGTGCGGACCCGTATGAGGTCTCCCTGCTGTACTGGGCATATTACGTTTCTGAGTGCGAGGGTATTCAGAGCCTC
ATGGGTACACGTGACGGTGCACAGTGGGCGTGGTGGTTTGGCGGCGCCGCGCAAGTGAGCTGGCGTATCGCGGAT
GCAATCGGCCGTGACAAATTCTTGCTGGAGTGGCCGGTGGATCGTATTGAGCACGACGAGAGCGGTGTGACCTTG
TTTAGCGGTCAACGTTCTCTGCGTGCGCGTCATATTGTGATCGCCATGAGCCCGCTGGCGGCGAACCAAATTCGT
TTCGAACCGGCGTTACCGACCTCCCGTGCACAGCTGCAGGCCCGTGCGCCGATGGGTCGTTACTATAAGGTGCAG
GCACGTTACCCGTCATCCTTCTGGGTTGAACAGGGCTACAGCGGTGCTCTGTTGGACACCGAAGATGTAGGTGTT
TTTCTGTTAGACGGCACGAAACCAACCGATACCTTGGCGACTCTCATTGGTTTCATCGGTGGTAGCAATTATGAT
CGCTGGGCTGCACACACCCCGCAAGAGCGCGAACGCGCGTTCCTGGACCTCCTGGTGAAAGCCTTCGGTCCGCAG
GCGGCGGACCCGAGCTATTTCCACGAAACGGATTGGACCCAACAAGAGTGGGCGAAGGGCGGCCCAGTTACCTAC
ATGCCGCCGGGTGTCCTTGCGAACTTCGGCGCAGCTCTTCGCGATCCGGTTGGCAAGGTGCATTTTGCAGGCACC
GAAGCCTCGTTCCAGTGGAGCGGCTACATGGAAGGTGGTGTGCGTGCAGGTCAGAAAGCTGCGGCTGCGATCGCC
GAGGAGTTGGAACGCACTGCTAACAAAGGCGCTTTAGTGTAA

>*Pp*DPKA
ATGAGTGCTCCCTCTACATCAACTGTAGTTCGCGTTCCGTTCACCGAACTGCAAAGCCTGCTACAAGCTATTTTT
CAGCGTCATGGTTGTTCGGAAGCGGTTGCGCGCGTGCTGGCGCACAACTGCGCGTCTGCCCAACGCGATGGAGCG
CACAGCCACGGCGTGTTTCGTATGCCGGGCTACGTTTCCACCCTGGCAAGCGGTTGGGTTGACGGCCAGGCAACG
CCGCAGGTTAGCGACGTGGCTGCTGGCTACGTGCGCGTCGACGCTGCAGGCGGCTTTGCACAGCCGGCGTTGGCA
GCGGCGAGAGAATTGTTGGTTGCCAAGGCGCGTTCTGCAGGCATCGCGGTGCTGGCGATCCACAACAGCCATCAT

TTCGCTGCTCTGTGGCCAGATGTGGAGCCGTTCGCGGAGGAGGGTCTGGTGGCTTTGTCCGTGGTTAATAGCATG
ACGTGCGTTGTCCCGCATGGTGCGCGTAAACCGCTCTTCGGTACAAATCCGATCGCCTTTGCGGCTCCGTGCGCG
GAACACGATCCGATTGTTTTCGACATGGCGACGAGCGCTATGGCACACGGCGATGTACAGATTGCGGCTCGCGCA
GGTCAGCAGCTGCCAGAAGGTATGGGTGTGGACGCCGATGGCCAACCGACCACCGATCCGAAAGCCATCCTGGAA
GGCGGCGCGCTGCTGCCGTTCGGCGGCCACAAGGGTTCCGCGCTTAGCATGATGGTTGAGTTACTGGCGGCGGCA
TTAACTGGTGGCCATTTCTCTTGGGAATTTGACTGGTCCGGTCATCCGGGTGCCAAGACCCCGTGGACCGGTCAA
CTGATTATCGTGATCAACCCGGGTAAGGCAGAGGGCGAACGTTTTGCGCAACGTAGCCGTGAGTTGGTCGAGCAC
ATGCAGGCGGTTGGTCTGACCCGTATGCCTGGTGAGCGTCGTTATCGTGAGCGCGAGGTGGCCGAAGAGGAGGGG
GTTGCGGTCACCGAACAGGAGTTACAAGGTCTGAAAGAATTGCTGGGTTAA

Section S4. HPLC, GC-MS analysis, methods, and columns

Table S6. HPLC Product analysis: methods and columns. Biotransformation reactions reductive amination of 4-phenyl-2-butanone and 1-indanone with methylamine were analysed by normal-phase chiral HPLC using Daicel columns: CHIRALPAK®IC 250 mm × 4.6 mm, 5 µm; and CHIRALPAK® IB N-5, 250 mm × 4.6 mm, 5 µm. Samples were run at 1 mL min^{-1} and monitored at a wavelength of 265 nm.

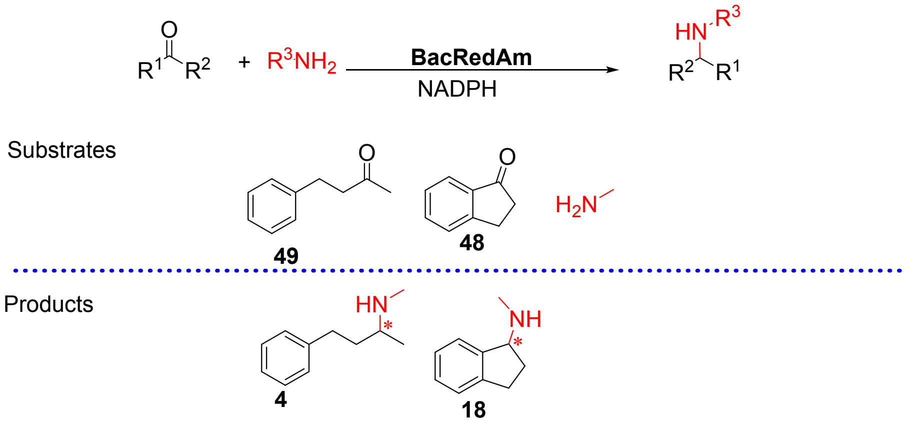

Method : *n*-hexane/isopropanol/diethylamine (98/02/0.1)

Substrates		Amine product	Column	Retention time		
				Ketone	Amine product	
Carbonyl	Amine				T1	T2
**49**	$MeNH_2$	**4**	CHIRALPAK®IB N-5	6.3	5.4 (*R*)	5.9 (*S*)
**48**	$MeNH_2$	**18**	CHIRALPAK®IC	21.4	8.2 (*S*)	8.6 (*R*)

Tables S7. GC-MS methods and retention time for analysis of RedAm-catalysed reductive amination of cyclohexanone**.**

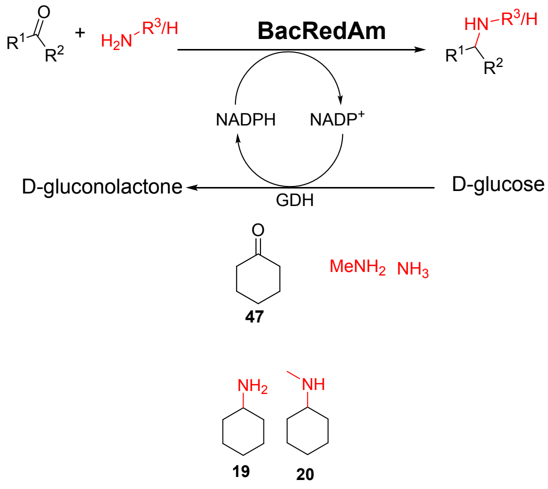

Column: CD-5MS Capillary Column 30 m x 0.25 mm x 0.25μm (P/N MOD-GC-CD5MSU-5, S/N CD17506. Method: inlet temperature = 250°C, detector temperature = 250°C, MS source= 230 °C, gas flow = 1.0 mL min^{-1}, oven temperature between 60 - 280°C, 20°C min^{-1} or 15 °C min^{-1}.

Ketone	Amine donor	Amine product	Ketone retention time [min]	Amine retention time [min]
**47**	Ammonia	**19**	6.2	5.7
**47**	methylamine	**20**	6.2	6.7

Tables S8. Rp-HPLC: methods and retention time for analysis of AmDH-catalysed reductive amination of cyclohexanone**.**

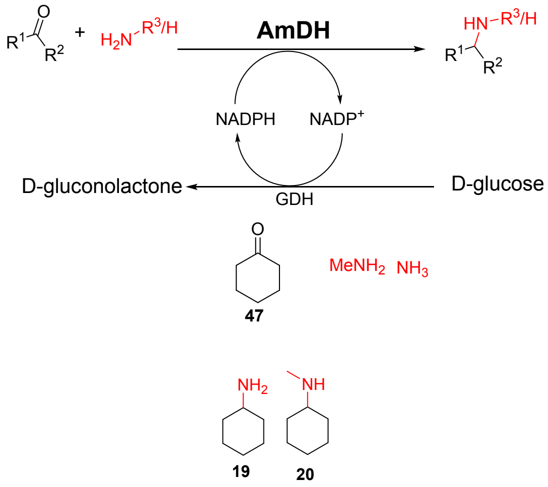

Method: *Water* + 0.1% formic acid *(A)*/Acetonitrile + 0.1% formic acid (B) (55/60/)- hold for 6 mins, then solvent B (acetonitrile) increase 60% over 8 mins, then solvent B (acetonitrile) reduce to 55% over 1 min, hold for 2 mins. Flow rate : 0.8 ml min^{-1}

Column: ZORBAX RR Eclipse Plus C18 Column, 4.6 x 150 mm, 3.5 μm.

Ketone	Amine donor	Amine product	Ketone retention time [min]	Amine (derivatised) retention time [min]
**47**	Ammonia	**19**		5.20
**47**	methylamine	**20**		5.73

Rp-HPLC chromatogram for analysis of *Am*DH-catalysed reactions.

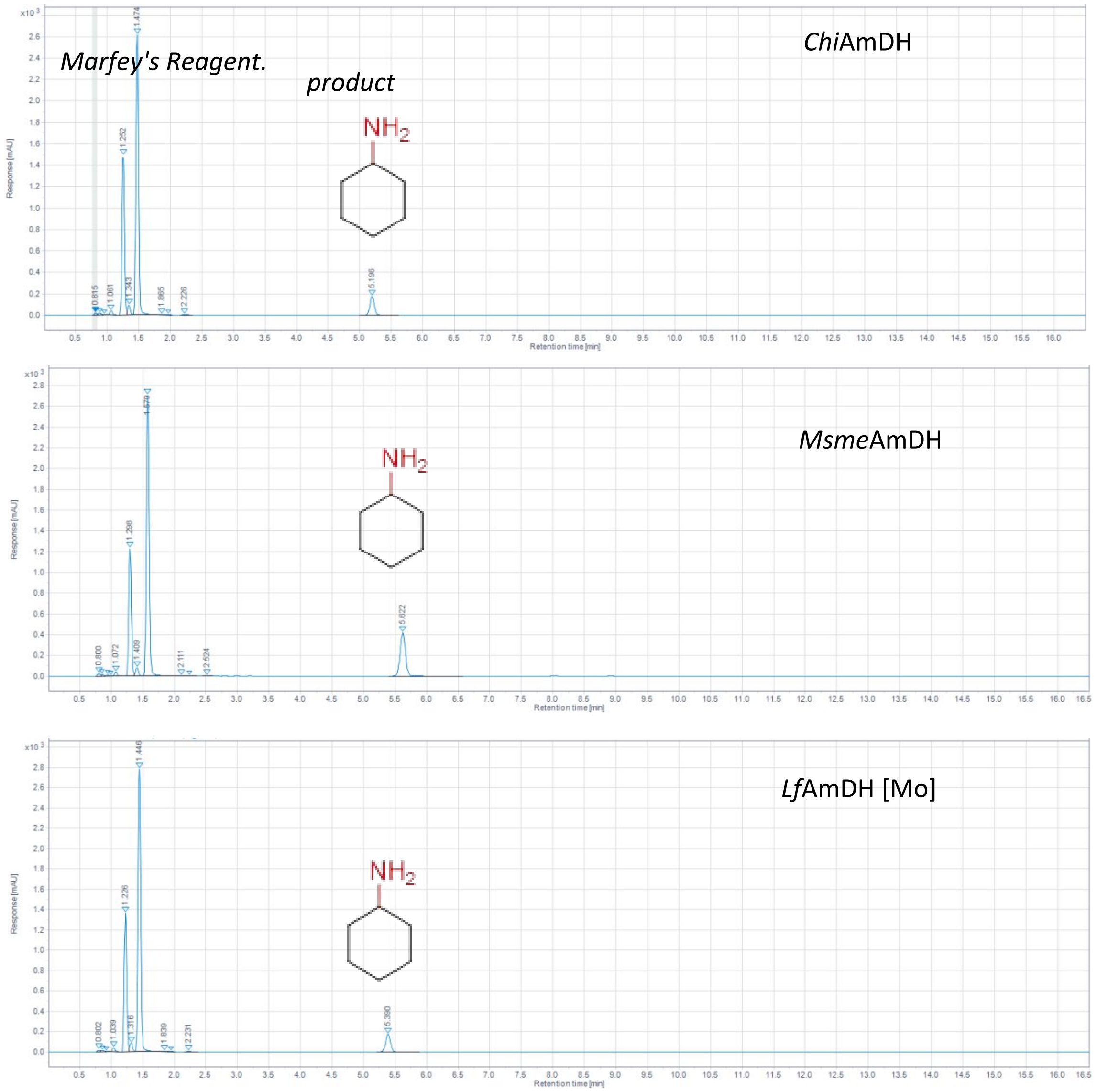

Figure S2. HPLC analysis biotransformation reactions for the reductive amination of cyclohexanone with ammonia, catalysed by ChiAmDH (top), MsmeAmDH (middle) and *Lf*AmDH [Mo]- bottom. Biotransformation was quenched with acetonitrile, derivatised with Marfey's reagent, and analysed on reverse HPLC.

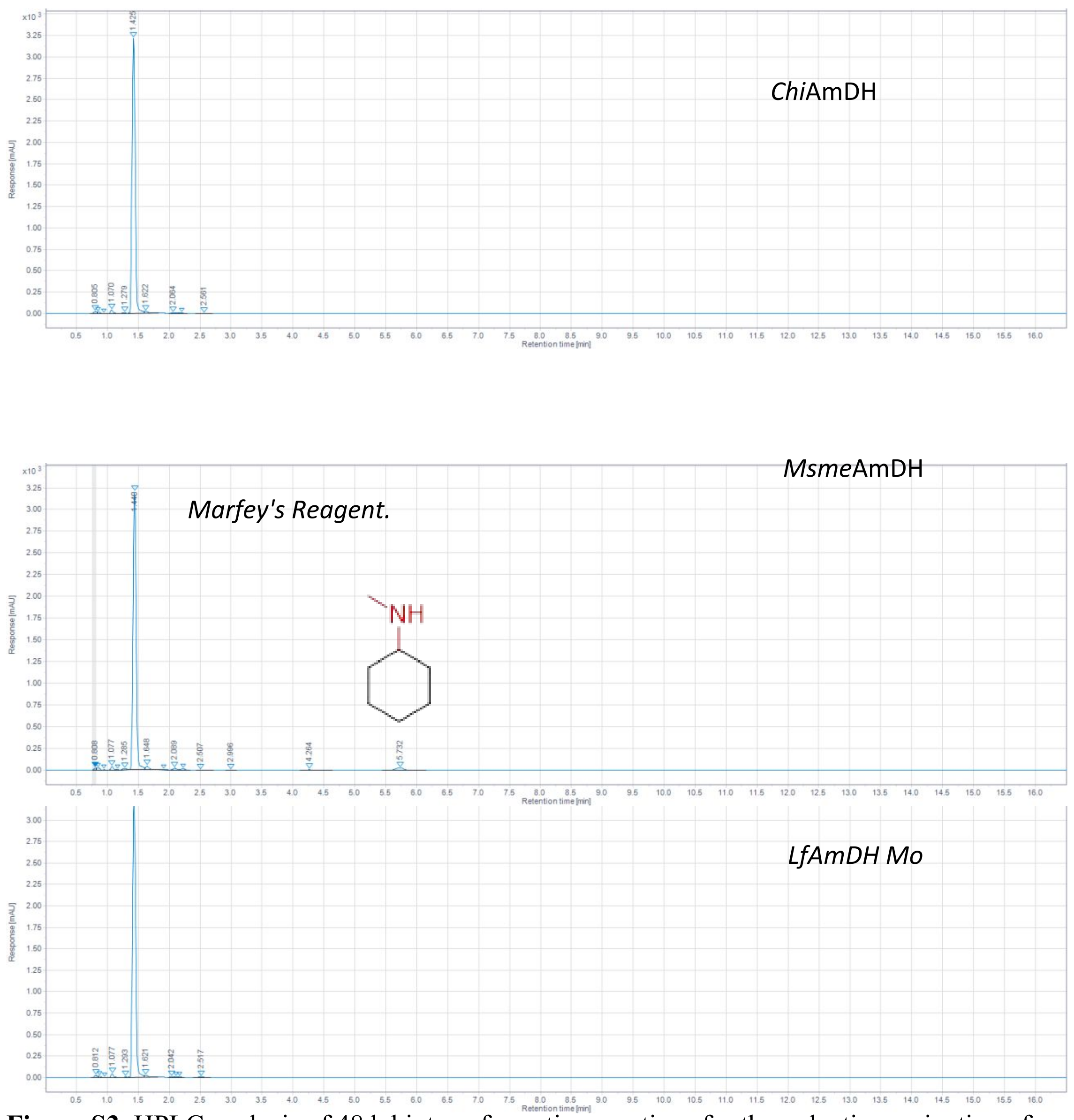

Figure S3. HPLC analysis of 48 h biotransformation reactions for the reductive amination of cyclohexanone with methylamine, catalysed by ChiAmDH (top), MsmeAmDH (middle), and *Lf*AmDH [Mo]. Biotransformation was quenched with acetonitrile, derivatised with Marfey's reagent, and analysed on reverse HPLC.

Supplementary References

(1) Ghislieri, D.; Green, A. P.; Pontini, M.; Willies, S. C.; Rowles, I.; Frank, A.; Grogan, G.; Turner, N. J. Engineering an Enantioselective Amine Oxidase for the Synthesis of Pharmaceutical Building Blocks and Alkaloid Natural Products. *J. Am. Chem. Soc.* **2013**, *135* (29), 10863–10869. https://doi.org/10.1021/ja4051235.

(2) DeMott, E.; Dickinson, D. J.; Doonan, R. Highly Improved Cloning Efficiency for Plasmid-Based CRISPR Knock-in in C. Elegans. *MicroPublication Biol. 2021*, 10.17912/micropub.biology.000499. https://doi.org/10.17912/micropub.biology.000499.

(3) Kim, G.; Lee, S.; Levy Karin, E.; Kim, H.; Moriwaki, Y.; Ovchinnikov, S.; Steinegger, M.; Mirdita, M. Easy and Accurate Protein Structure Prediction Using ColabFold. *Nat. Protoc.* **2024**, 1–23. https://doi.org/10.1038/s41596-024-01060-5.

(4) Hekkelman, M. L.; de Vries, I.; Joosten, R. P.; Perrakis, A. AlphaFill: Enriching AlphaFold Models with Ligands and Cofactors. *Nat. Methods* **2023**, *20* (2), 205–213. https://doi.org/10.1038/s41592-022-01685-y.

(5) Krieger, E.; Joo, K.; Lee, J.; Lee, J.; Raman, S.; Thompson, J.; Tyka, M.; Baker, D.; Karplus, K. Improving Physical Realism, Stereochemistry, and Side-Chain Accuracy in Homology Modeling: Four Approaches That Performed Well in CASP8. *Proteins Struct. Funct. Bioinforma.* **2009**, *77* (S9), 114–122. https://doi.org/10.1002/prot.22570.

(6) Schober, M.; MacDermaid, C.; Ollis, A. A.; Chang, S.; Khan, D.; Hosford, J.; Latham, J.; Ihnken, L. A. F.; Brown, M. J. B.; Fuerst, D.; Sanganee, M. J.; Roiban, G.-D. Chiral Synthesis of LSD1 Inhibitor GSK2879552 Enabled by Directed Evolution of an Imine Reductase. *Nat. Catal.* **2019**, *2* (10), 909–915. https://doi.org/10.1038/s41929-019-0341-4.

(7) Kumar, R.; Karmilowicz, M. J.; Burke, D.; Burns, M. P.; Clark, L. A.; Connor, C. G.; Cordi, E.; Do, N. M.; Doyle, K. M.; Hoagland, S.; Lewis, C. A.; Mangan, D.; Martinez, C. A.; McInturff, E. L.; Meldrum, K.; Pearson, R.; Steflik, J.; Rane, A.; Weaver, J. Biocatalytic Reductive Amination from Discovery to Commercial Manufacturing Applied to Abrocitinib JAK1 Inhibitor. *Nat. Catal.* **2021**, *4* (9), 775–782. https://doi.org/10.1038/s41929-021-00671-5.

(8) Steflik, J.; Gilio, A.; Burns, M.; Grogan, G.; Kumar, R.; Lewis, R.; Martinez, C. Engineering of a Reductive Aminase to Enable the Synthesis of a Key Intermediate to a CDK 2/4/6 Inhibitor. *ACS Catal.* **2023**, *13* (15), 10065–10075. https://doi.org/10.1021/acscatal.3c01534.
